# Limited impact of the siRNA pathway on transposable element expression in *Aedes aegypti*

**DOI:** 10.1186/s12915-025-02225-8

**Published:** 2025-05-13

**Authors:** Alexander Bergman, Anna B. Crist, Hélène Lopez-Maestre, Hervé Blanc, Mauro Castelló-Sanjuán, Lionel Frangeul, Hugo Varet, Josquin Daron, Sarah H. Merkling, Maria-Carla Saleh, Louis Lambrechts

**Affiliations:** 1 Institut Pasteur, Université Paris Cité, CNRS UMR2000, Insect-Virus Interactions Unit, Paris, France; 2 Institut Pasteur, Université Paris Cité, Bioinformatics and Biostatistics Hub, Paris, France; 3 Institut Pasteur, Université Paris Cité, Viruses and RNA Interference Unit, Paris, France

**Keywords:** Mosquito, *Aedes aegypti*, *Dicer2*, Transposable element, siRNA pathway, piRNA pathway

## Abstract

**Background:**

Transposable elements (TEs) are DNA sequences that can change their position within a genome. In insects, small RNA pathways are central to the transcriptional and post-transcriptional regulation of TE expression. The Piwi-interacting RNA (piRNA) pathway is particularly important in germline tissues, where it silences TE transcripts via small RNAs of 24–30 nucleotides (nt) in length produced from genomic precursor transcripts as well as through a “ping-pong” amplification cycle. The small interfering RNA (siRNA) pathway helps restrict TE expression in somatic tissues via 21nt small RNAs produced from double-stranded RNA by the endonuclease Dicer2, which guide an RNA-induced silencing complex to degrade complementary RNAs. However, much of this knowledge comes from studies of the model insect *Drosophila melanogaster*. In the mosquito *Aedes aegypti*, a medically significant vector species, the siRNA pathway has mainly been investigated in connection with its antiviral role, leaving open whether it also regulates TE expression.

**Results:**

We investigated the expression of TEs and small RNAs in both somatic and gonadal tissues of a *Dicer2* mutant line of *Ae. aegypti* and its wild-type counterpart. Our results show a modified pattern of TE expression and a decrease in TE-derived 21nt RNAs in the *Dicer2* mutant, but no major shift of TE transcript abundance. The lack of a functional siRNA pathway also causes perturbations in piRNA ping-pong signatures and the expression of certain piRNA-associated genes, but without clear evidence for compensation by increased piRNA pathway activity.

**Conclusions:**

The mosquito *Ae. aegypti* produces siRNAs derived from TEs but these lack a critical role in the regulation of TE expression both in somatic and in gonadal tissues.

**Supplementary Information:**

The online version contains supplementary material available at 10.1186/s12915-025-02225-8.

## Background

Transposable elements (TEs), also known as transposons, are DNA sequences capable of moving within genomes [1]. With a few notable exceptions, such as some apicomplexan parasites, TEs have been found in nearly all eukaryotic genomes in widely varying proportions [2]. Among dipteran insect species, TEs have also had variable evolutionary success. The genome of the model organism *Drosophila melanogaster* is relatively poor in TEs, with a TE genome fraction of only 20% [3]. Among mosquitoes, the proportion of TEs in the genome is substantially higher for several species of the Culicinae subfamily, with TE genome fractions above 40% [4–7] and even over 60% for *Aedes aegypti*, compared to < 20% in the Anophelinae subfamily [4, 8, 9].

Depending on the presence or absence of an RNA intermediate in the transposition mechanism, TEs are divided into two classes—class I and class II. TEs that do have an RNA intermediate, such as long terminal repeat (LTR) transposons, long interspersed nuclear elements (LINEs), and short interspersed nuclear elements (SINEs) are designated class I or retrotransposons. They rely on either a self-encoded or, as is the case for SINEs, a stray reverse transcriptase (RT) to complete their transposition cycle. Class II TEs, also known as DNA transposons, such as terminal inverted repeats (TIR) transposons and helitrons, lack an RNA intermediate and transpose through a “cut-and-paste” mechanism. Nonetheless, the expression of encoded proteins (e.g., transposase), allows for detection of autonomous TIR transposon expression at the RNA level, as opposed to non-autonomous TIR transposons, such as miniature inverted repeat TEs (MITEs), which only exist in DNA form [10].

Due to the potentially deleterious effects of rogue transposition on genomic organization and stability, organisms have evolved various strategies to repress TE expression, such as small RNA pathways, which carry out both transcriptional and post-transcriptional silencing of TEs [11–14]. In *D. melanogaster*, TE expression in the germline and surrounding ovarian tissues is regulated by the Piwi-interacting RNA (piRNA) pathway. piRNAs are small RNAs of 24–30 nucleotides (nt) in length generated from genomic precursors as well as through a “ping-pong” amplification loop, in which secondary piRNAs are generated through the cleavage of TE transcripts [15, 16]. In differentiated, non-gonadal somatic tissues, where piRNAs in *D. melanogaster* have been elusive, the piRNA pathway may indirectly regulate somatic TE expression through a redundant two-layer mechanism involving embryonic piRNA-mediated silencing and a small interfering RNA (siRNA)-dependent backup [17]. In flies, siRNAs are 21nt RNAs generated from double-stranded RNA (dsRNA) by the endonuclease Dicer2, guiding an RNA-induced silencing complex (RISC) to target a complementary RNA for degradation [18, 19]. The siRNA pathway by itself has also been shown to regulate TE expression in somatic tissues using endogenous siRNAs (endo-siRNAs) [20–23]. Most of our knowledge about how small RNA pathways affect TE expression comes from studies in the model insect *D. melanogaster*, and whether these findings can be extended to other dipteran insects remains largely unknown. Small RNA silencing pathways have been repurposed for both somatic and germline functions throughout arthropod evolution [24].

The yellow fever mosquito, *Aedes aegypti*, is an infamous vector of multiple arthropod-borne viruses (arboviruses) of medical significance, such as dengue, Zika, and chikungunya viruses [25–27]. Due to the major impact of this species on human health, the role of the siRNA pathway in *Ae. aegypti* has been previously studied in light of its antiviral function [28–33]. However, whether the siRNA pathway also regulates TEs in this species, which also has abundant somatic piRNAs [24, 34, 35], remains unclear. Using a *Dicer2* (*Dcr2*) mutant line [32] and an improved annotation of TEs in *Ae. aegypti* [36], we analyzed the transcriptomic and small RNA landscapes of the midguts and ovaries of the mutant and its wild-type control. Our findings suggest that although *Ae. aegypti* produces endo-siRNAs derived from both TEs and genes, the endogenous siRNA pathway has an overall limited effect on TE and gene expression.

## Results

To determine the role of the siRNA pathway in the regulation of TE expression in *Ae. aegypti*, we analyzed the abundance of TE transcripts and small RNAs in the midgut and the ovaries of the *Dcr2* mutant and its wild-type control. We excluded non-autonomous and unknown TEs from the main analysis due to frequent chimerism with gene transcripts (Additional file 1: Fig. S1) and limited significance. Nonetheless, we provide the full results in Additional files 2–10: Fig. S2, Fig. S3, Fig. S4, Fig. S5, Fig. S6, Fig. S7, Fig. S8, Fig. S9, and Fig. S10.

### *Dcr2* mutant displays specific differences but no uniform shift in TE expression

**Fig. 1:**
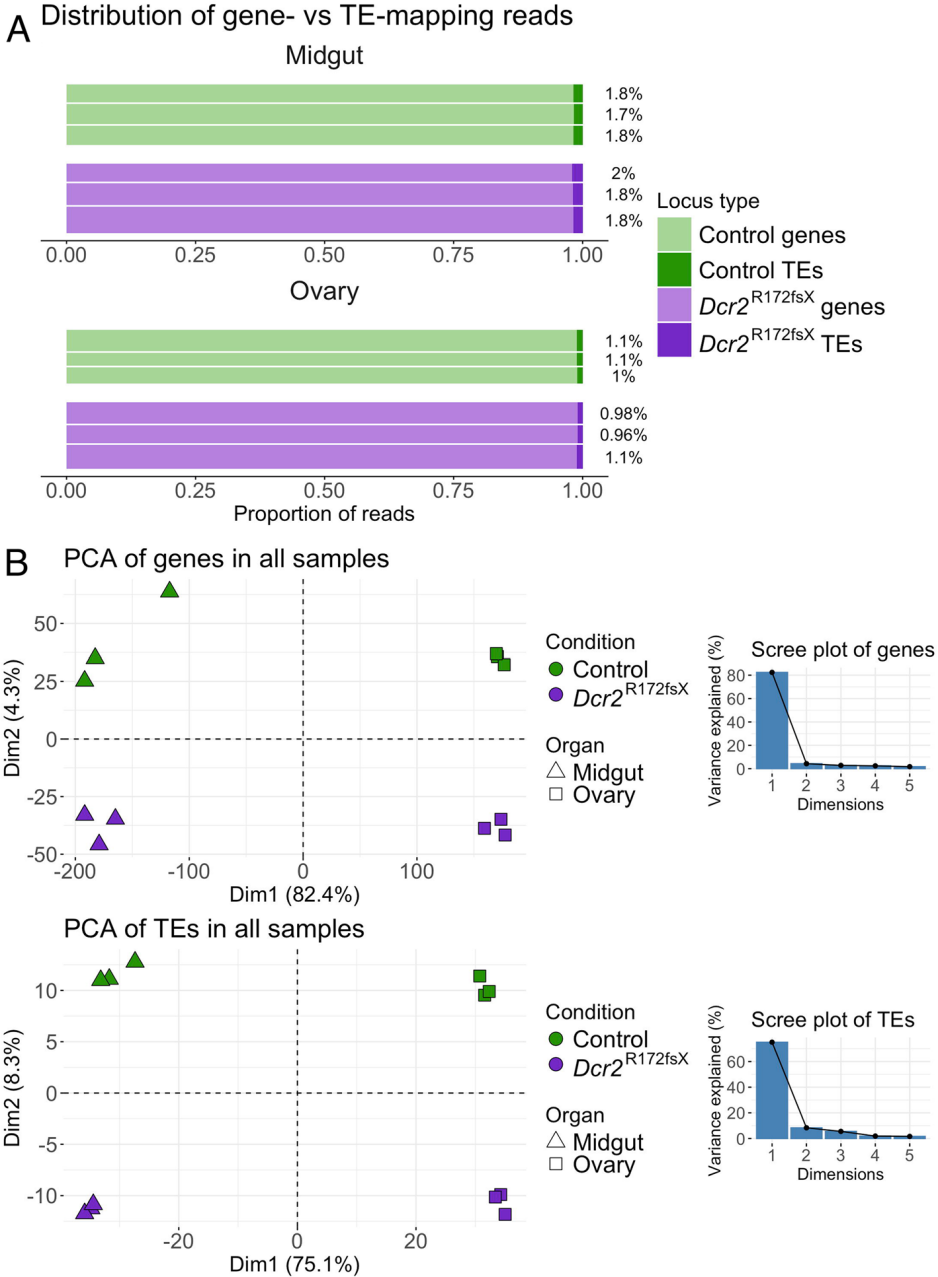
TE and gene expression patterns differ in the *Dcr2* mutant. **A** Proportion of reads mapping to either genes or TEs in the RNA-seq libraries from midguts (top graph) and ovaries (bottom graph). The vertical height of the bar is proportional to library size (number of counted reads). Percentages of TE-mapping reads are stated on the right side. **B** Separate principal component analyses (PCAs) of gene (top graph) and TE (bottom graph) read counts with accompanying scree plots (line plots of the eigenvalues of principal components)

We first compared the abundance of gene- and TE-derived transcripts between the *Dcr2* mutant and the wild-type control. In total, the RNA-sequencing (RNA-seq) libraries yielded between 15.1 million and 30.6 million read counts per biological replicate. Of these, approximately 2% in midgut samples and 1% in ovary samples originated from TEs (Fig. 1A). The proportion of TE counts was significantly influenced by the organ but not by the mosquito line (analysis of variance on logit-transformed proportions, *p* = 0.79 for line, *p* = 6.4 × 10^−8^ for organ, non-significant interaction excluded from the model). A PCA confirmed the clear distinction between the tissues in terms of both gene and TE expression, with the first component explaining 82 and 75% of the variation, respectively (Fig. 1B). However, the PCA also showed a clear separation of the two conditions along the second axis, which explained 4.3% of the variation for genes and 8.3% of the variation for TEs.

**Fig. 2:**
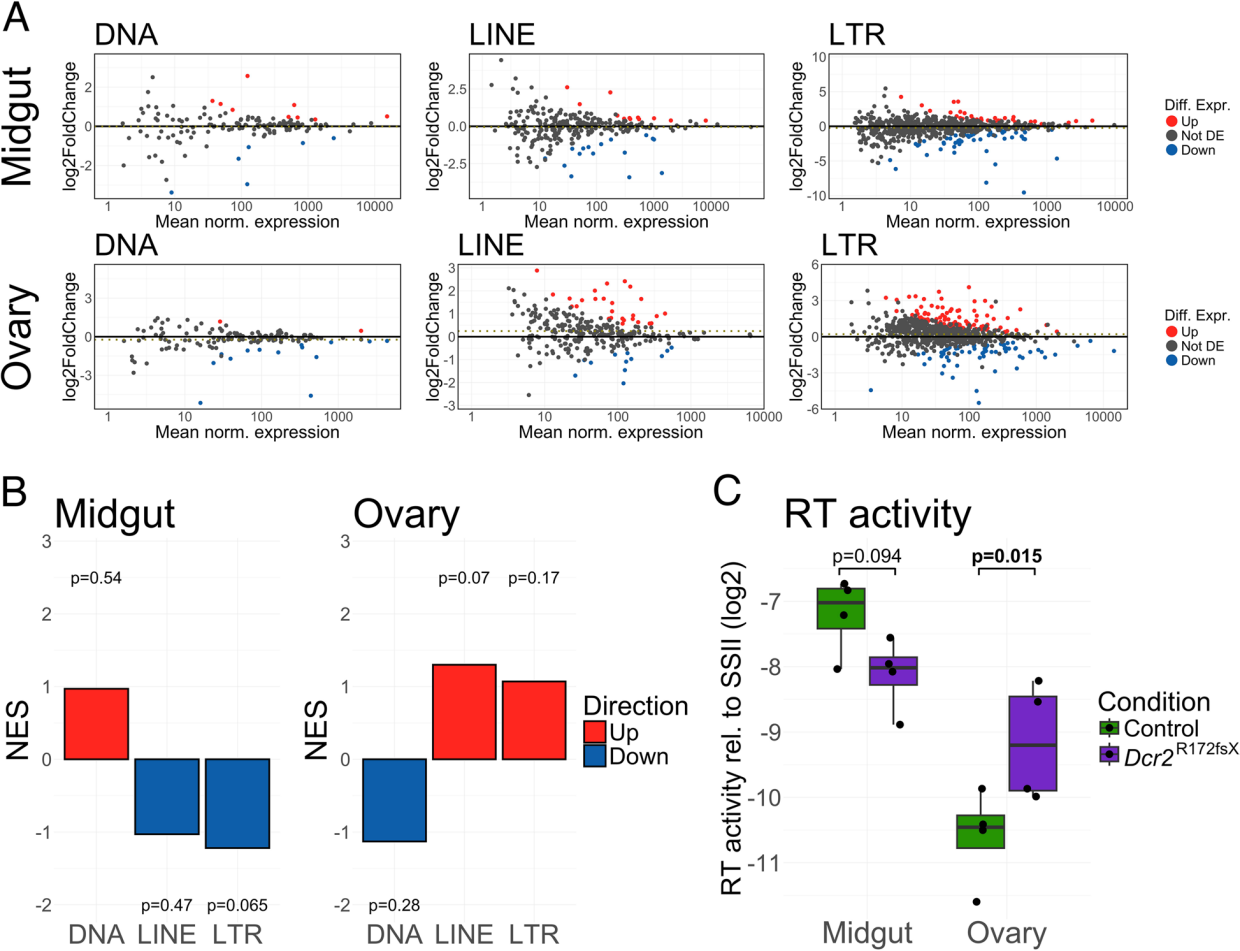
TE expression in the *Dcr2* mutant is perturbed but not uniformly shifted. **A** MA plots of individual TE families grouped by order (DNA, LINE, LTR) for midguts (top row) and ovaries (bottom row). The *x*-axis shows mean read counts normalized by the median of ratios (DESeq2-based normalization) and the *y*-axis shows log_2_ fold-change in the *Dcr2* mutant line. Families are colored according to their differential expression (red: enriched in mutant line; blue: depleted in mutant line; grey: not differentially expressed). The dotted line in the center of each plot represents the mean log_2_ fold-change. **B** Gene set enrichment analysis (GSEA) results. The height of each bar represents the normalized enrichment score (NES), i.e., the relative, rank-based enrichment of the TE order compared to a random, equally sized, group of transcripts. *P*-values above or below the bars indicate the false discovery rate for the enrichment (red bars) or depletion (blue bars) in the *Dcr2* mutant relative to the wild-type control. **C** Box plot of RT activity measured in midgut and ovary samples relative to SuperScript II (SSII). *P*-values shown above the graph were generated by pairwise comparisons within a linear mixed effect model using Wald’s test

Differential expression analysis revealed that specific TE families were both enriched and depleted between the lines for every order of autonomous TEs (Fig. 2A). In the midgut, 24 families were enriched and 60 families were depleted with an absolute log_2_ fold-change > 1 at a significance level of adjusted *p* < 0.05. Under the same criteria, 82 families were enriched and 57 depleted in the ovaries (Additional file 11: Table S1). Nonetheless, gene set enrichment analysis (GSEA) found that no TE order was differentially expressed as a whole (Fig. 2B). Functional validation using an RT activity assay followed the trends seen in the GSEA analysis for LINE and LTR transposons, with a trend toward lower RT activity in the midgut and higher RT activity in the ovaries (Fig. 2C). The linear mixed effect model used to analyze RT activity data (Fig. 2C) showed a significant interaction between organ and line (*p* = 0.008), reflecting the opposite effects of the *Dcr2* mutation on RT activity in the midgut and the ovary. Together, these data provide evidence that differences in the expression of specific TE families do exist in the absence of *Dcr2*, but they are not consistent across TE orders or tissues in their direction or their magnitude.

To corroborate our data with a different siRNA pathway mutant, we re-analyzed the public RNA-seq dataset of an *Ae. aegypti Ago2* mutant line by Dong and Dimopoulos [31]. In this mutant, we saw significant enrichment of multiple orders of TEs, including DNA and LTR transposons (Additional file 2: Fig. S2). As for corroboration of previous findings in *D. melanogaster*, we re-analyzed two publicly available RNA-seq datasets from gonadal and somatic tissues of *Dcr2* mutants [17, 37]. We found that even in *D. melanogaster*, the lack of Dicer2 does not lead to generalized TE deregulation (Additional file 2: Fig. S2). Mutating both *Dcr2* and *Piwi*, however, clearly leads to dramatic enrichment of transcripts from all TE orders (Additional file 2: Fig. S2).

### Reduction of TE-derived 21nt RNAs does not correlate with expression differences

**Fig. 3:**
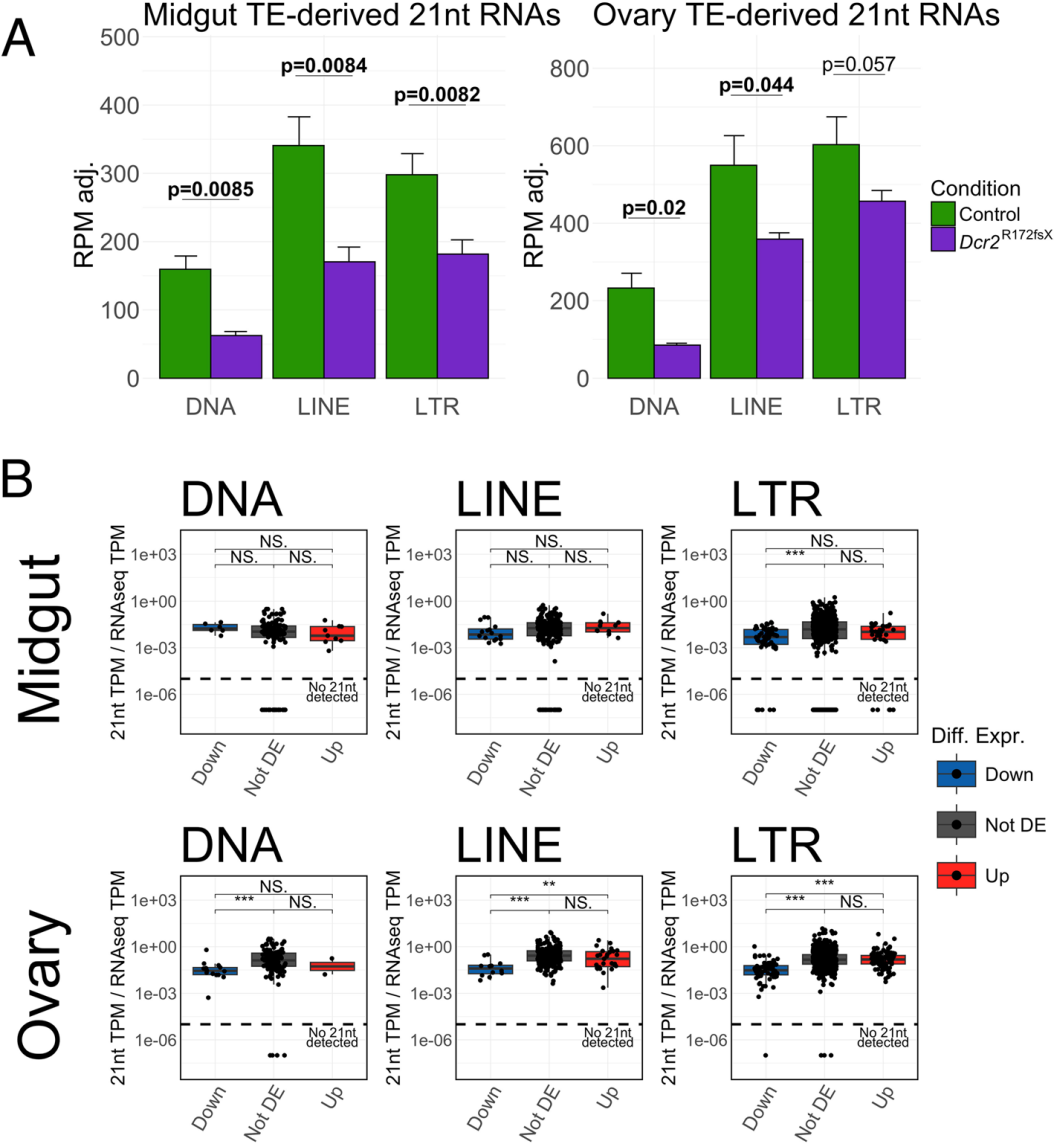
Reduction of TE-derived 21nt RNAs in the *Dcr2* mutant does not correlate with differential TE expression. **A** miRNA-adjusted reads per million mapped reads (RPM) mapping to the different TE orders in the midgut (left plot) and ovaries (right plot) of *Dcr2* mutant and control mosquitoes. Error bars denote one standard deviation. *P*-values shown above the bars were generated with Welch’s *t*-test. **B** Ratios between miRNA-adjusted 21nt RNAs expressed in transcripts per million (TPM) and RNA-seq TPM in the control mosquitoes for individual TE families depleted (Down), non-differentially expressed (Not DE), and enriched (Up) in the *Dcr2* mutant within each TE order for midguts (top row) and ovaries (bottom row). *P*-values shown above the graphs were generated using Wilcoxon rank sum test (*p < 0.05, **p < 0.01, ***p < 0.001, NS = non-significant). Families with no detected transcripts were excluded. Families with detected transcripts but no 21nt RNAs detected are shown below the dashed line

To determine whether the lack of a major shift in TE transcript abundance in the absence of *Dcr2* was simply due to a general lack of TE-derived siRNAs also in the wild-type condition, we examined the abundance of TE-derived 21nt RNAs. We mapped small RNAs to the genome with random attribution of multimapping reads to retain the correct total number of reads. Misattribution through randomization was rare, with at most 1.5 and 3.1% of multimapping 21nt reads mapping to more than one TE family in midgut and ovary samples, respectively. We scaled reads per million mapped reads (RPM) values by total miRNA RPM to adjust for the change in proportion following a change in composition of small RNA-seq data, following the assumption of a constant size of the miRNA pool. In the *Dcr2* mutant, all autonomous TE orders exhibited a significant reduction in the abundance of TE-mapping 21nt RNAs except LTR transposons in the ovaries, for which the difference was marginally non-significant (Fig. 3A). This finding was confirmed in the re-analysis of published whole-mosquito small RNA-seq data from an independent *Dcr2* mutant [33], with substantial reductions in 21nt RNAs mapping to all orders of TEs (Additional file 3: Fig. S3). Together, these results confirm that TE transcripts act as a source of siRNAs in *Ae. aegypti*.

To explain the discrepancy between the overall depletion of siRNAs and the lack of a major shift in TE transcript abundance in the *Dcr2* mutant, we hypothesized that only some TE families within each order were disproportionately affected by a dysfunctional siRNA pathway. Under this hypothesis, we predicted that the TE families with the greatest abundance of 21nt RNAs (i.e., TE families most implicated in siRNA biogenesis) in the wild-type line would display the highest enrichment in the *Dcr2* mutant. The analysis of ratios between 21nt RNA and transcript abundances by TE family did not support this hypothesis. Instead, we found that TE families whose expression was depleted in the *Dcr2* mutant line tended to display the lowest amounts of TE-derived 21nt RNAs in the wild-type line (Fig. 3B). The families enriched in the *Dcr2* mutant were thus not those producing the most abundant 21nt RNAs in the wild-type control. Overall, we found that in both the midgut and the ovaries, fewer TE-derived 21nt RNAs were detected in the *Dcr2* mutant but the reduction did not correlate with differential TE expression.

### Lack of *Dcr2* only causes a minor change in piRNA ping-pong activity in the midgut

**Fig. 4:**
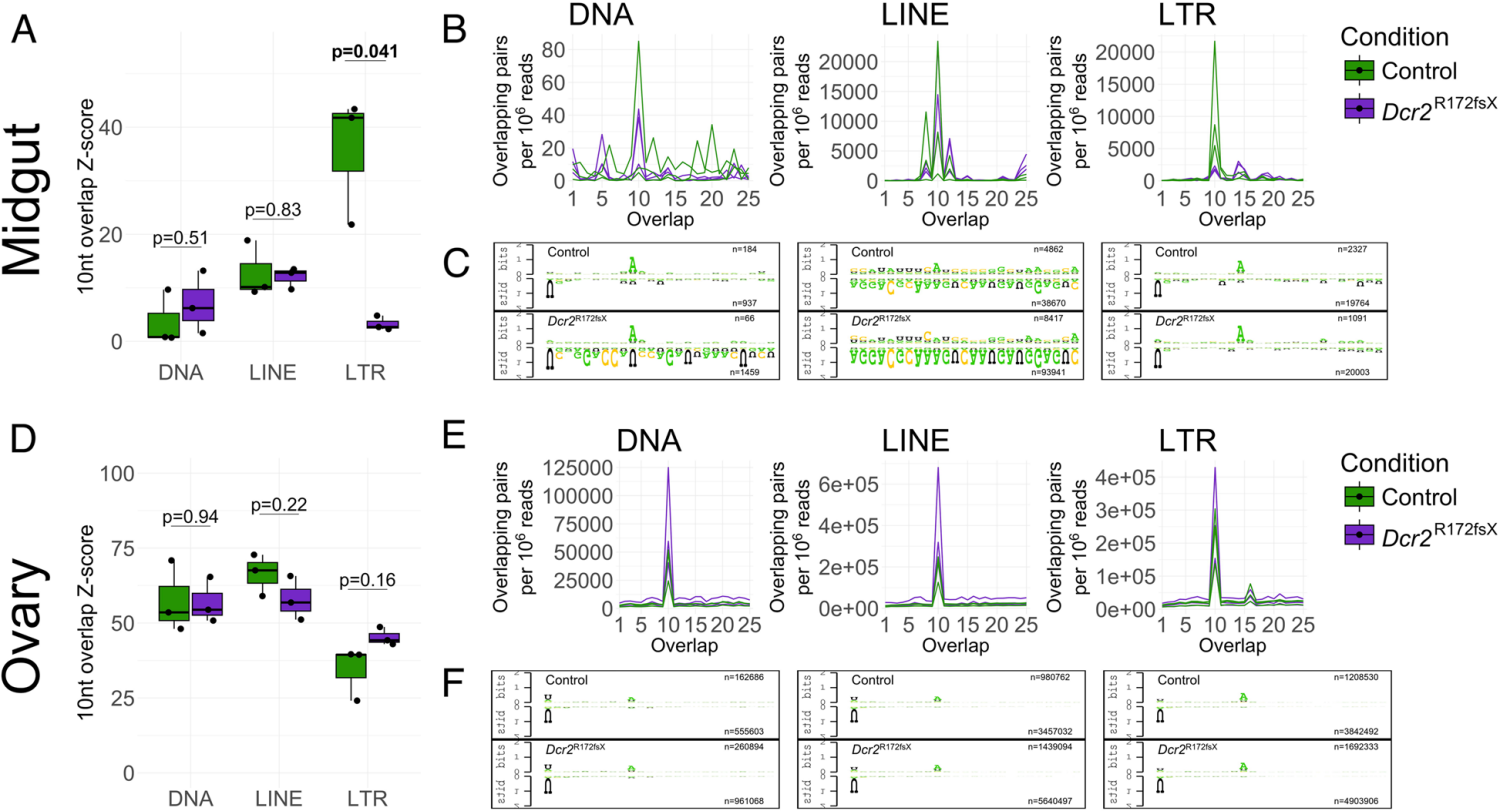
TE-derived piRNAs display mostly unchanged ping-pong activity in the *Dcr2* mutant. **A**, **D** Box plots of 10nt overlap *Z*-scores among 26–30nt sense and antisense reads mapping to TEs stratified by order in midguts (**A**) and ovaries (**D**) of the *Dcr2* mutant and the wild-type control. *P*-values indicated above the box pairs were generated using Welch’s *t*-test. Significant *p*-values are highlighted in bold font. **B**, **E** Frequency of overlaps among sense and antisense 26–30nt reads by a given number of nt for midguts (**B**) and ovaries (**E**). **C**, **F** Logo plots of sense (“secondary,” top logo) and antisense (“primary,” bottom logo) 26–30nt reads overlapping each other by 10nt for control (top row) and *Dcr2* mutant (bottom row) samples mapping to DNA (left plot), LINE (center plot), and LTR (right plot) transposons for midgut (**C**) and ovary (**F**). The sequences from all three replicates were merged into one logo and trimmed to 25 nt. The number of sequences used to construct the logos is specified on the right side within each plot. For complete and replicate-specific logo plots, see Additional file 12: Fig. S11 for autonomous TEs and Additional file 8: Fig. S8 for non-autonomous TEs

Since a reduction in TE-derived 21nt RNAs was not associated with a corresponding increase in transcript abundance, we investigated whether there was any compensation through increased ping-pong activity of the piRNA pathway. In the ovary, canonical signatures of ping-pong amplification could be observed for the three autonomous TE orders (DNA, LINE, and LTR) in both control and mutant mosquitoes, with both frequent 10nt overlaps and a corresponding 1U and 10 A bias in putative primary and secondary piRNAs, respectively (Fig. 4). In the midgut, evidence of ping-pong activity, consisting of frequent 10nt overlaps and 1U/10 A biases could only be seen for DNA and LTR transposons (Fig. 4). For DNA transposons, the overabundance of 10nt overlaps varied between biological replicates (Fig. 4). Furthermore, the number of putative secondary piRNAs was negligible (8–167 reads) and specific sequences dominated putative primary piRNAs involved in ping-pong amplification (Additional file 12: Fig. S11). The importance of the ping-pong cycle in DNA transposon regulation in the midgut is therefore likely minimal. The only significant difference that we observed between the two mosquito lines was a decrease in 10nt overlaps for LTR transposons in the midgut of the *Dcr2* mutant (Fig. 4). However, a 10 A and 1U bias was seen for LTR-derived putative piRNAs in both conditions (Fig. 4C), and no differences were observed in the amounts of putative secondary (Additional file 4: Fig. S4) nor primary (Additional file 5: Fig. S5) piRNAs. Closer inspection revealed that the vast majority of the piRNA pairs overlapping by 10nt in the wild-type mosquitoes originated from a single TE copy belonging to the family TE_0669_Gypsy (Additional file 6: Fig. S6), the secondary piRNA-component of which was mainly composed of a single sequence (Additional file 13: Fig. S12). Although 10nt overlaps from this family are also abundant in the *Dcr2* mutant, these originate from multiple other TE copies and do not account for as many of the 10nt-overlapping pairs (Additional file 6: Fig. S6). Removing this single locus from the analysis abrogated any difference between the two conditions (Additional file 7: Fig. S7). Furthermore, this analysis showed that the ping-pong activity for LTR transposons in the midgut was largely driven by a handful of Gypsy superfamily members (Additional file 6: Fig. S6 A), with ping-pong activity being low in the midgut in general (Additional files 4, 5, 7, 8 and 12: Fig. S4, Fig. S5, Fig. S7, Fig. S8, and Fig. S11). Overall, we only detected a change in ping-pong signatures for LTR transposons in the midgut, but this difference was attributable to a single locus. We found no generalized compensation by the piRNA pathway for the lack of *Dcr2*.

### Transcriptomic changes show no obvious compensatory mechanism in *Dcr2* mutant

To investigate whether the lack of *Dcr2* was associated with another compensatory mechanism than piRNA ping-pong activity that could contribute to TE expression regulation, we performed a differential gene expression analysis of the RNA-seq data. In total, 413 genes (127 enriched, 286 depleted) in the midgut and 1234 genes (546 enriched, 688 depleted) in the ovaries were differentially expressed in the *Dcr2* mutant with an absolute log_2_ fold-change > 1 below the significance threshold of an adjusted *p* < 0.05 (Additional files 9 and 11: Fig. S9 and Table S1). In the ovaries, several metabolic pathways were downregulated, while pathways related to nuclear processes, such as transcription factors and spliceosome-associated genes, were enriched. In the midgut, the only significantly differentially regulated pathway was nucleotide excision repair (Additional file 14: Fig. S13 A). A gene-by-gene analysis of the differential expression of siRNA-, piRNA-, and histone modification-related genes showed a depletion of certain factors involved in piRNA biogenesis, such as *Yb*, *papi*, and *vas* homologs, as well as *Piwi1/3* and *Piwi2* in the ovaries. Interestingly, expression of *Dicer1*, canonically an essential gene for miRNA biogenesis, was slightly elevated (Additional file 14: Fig. S13B). Overall, the *Dcr2* mutation caused significant perturbation in the transcriptome homeostasis of the ovaries, but no single compensatory mechanism could be discerned from our dataset.

To further investigate the relationship between a dysfunctional siRNA pathway and other conventional mechanisms for TE regulation, we extended our gene set enrichment analysis (GSEA) to the re-analyzed single mutant *Ae. aegypti* and *D. melanogaster* datasets. In the *Ae. aegypti Ago2* mutant line, our re-analysis showed significant depletions of both piRNA- and histone modification-related genes. For *D. melanogaster Dcr2* mutants, the two datasets produced different results for somatic tissues, with piRNA pathway genes being depleted in the carcasses (whole bodies without ovaries) and histone-modifying genes being depleted in the heads (Additional file 15: Fig. S14).

### Endo-siRNAs are of limited importance in gene regulation in *Ae. aegypti*

**Fig. 5:**
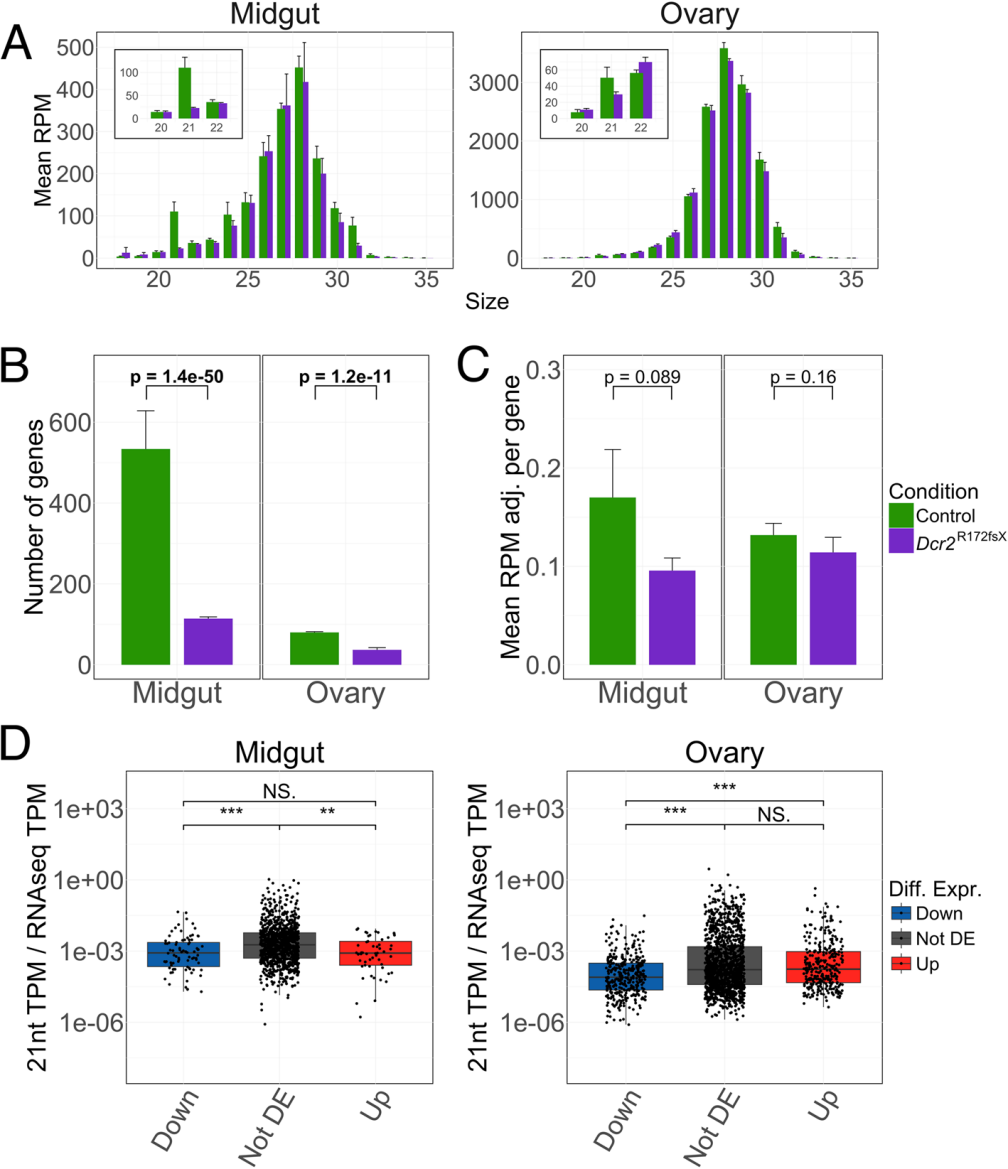
Gene-derived endo-siRNAs are detectable but have minor effects on gene expression. **A** Size distribution profiles for small RNA reads mapping uniquely and in the antisense orientation relative to gene exons in the midgut (right panel) and ovary (left panel). The *y*-axes show the reads per million mapped reads (RPM), which was calculated with the total number of mapped reads (i.e., both sense and antisense as well as non-uniquely mapping reads) as the denominator. The error bar denotes one standard deviation. An insert highlights the sizes immediately adjacent to 21 nt in the two mosquito lines. **B** Number of genes in each organ and condition with a whole number of read counts equivalent to 0.1 RPM or higher uniquely mapping to the antisense orientation relative to the annotated gene (i.e. exons thereof). For each bar, *n* = 3. The error bar denotes one standard deviation. Statistical significance was determined through a negative binomial generalized linear model for each organ separately. **C** Mean antisense- and uniquely mapping RPM of 21nt reads adjusted by total miRNA counts (RPM adj.) of the genes tallied in **B** for each organ and condition. Only the genes surpassing the 0.1 RPM-equivalence threshold in each biological replicate were included for the mean of that replicate. For each bar, *n* = 3. The error bar denotes one standard deviation. Statistical significance was determined using Welch’s *t*-test. **D** Ratios between miRNA-adjusted 21nt reads mapping uniquely in the antisense orientation expressed in transcripts per million (TPM) and RNA-seq TPM in the control mosquitoes for genes depleted (Down), non-differentially expressed (Not DE), and enriched (Up) in the *Dcr2* mutant within each TE order for midguts (left plot) and ovaries (right plot). *P*-values shown above the graphs were generated using Wilcoxon rank sum test (*p < 0.05, **p < 0.01, ***p < 0.001, NS = non-significant). Genes with no detected transcripts or antisense-mapping 21nt RNAs were excluded

To explore whether genes can act as a source of endo-siRNAs in *Ae. aegypti*, we counted reads mapping to unique positions in the antisense orientation to all annotated genes. Despite filtering the alignments for reads mapping to piRNA clusters, the size distribution profiles were dominated by piRNA-sized reads in both midgut and ovary (Fig. 5A). Nonetheless, a sharp peak at 21 nt observed in the midguts of wild-type mosquitoes was missing in the *Dcr2* mutant and the proportion of antisense-mapping 21nt RNAs was slightly lower in the ovaries as well (Fig. 5A). Furthermore, significantly more genes surpassed the 0.1 RPM threshold we set as a limit for acting as a source of endo-siRNAs in both the midguts and the ovaries of the control mosquitoes (Fig. 5B). The extent to which 21nt RNAs are produced per gene, measured as mean miRNA-adjusted RPM of antisense-mapping reads for every gene within each biological replicate, was, however, not significantly different between the two conditions (Fig. 5C). Taken together, these results suggest that endo-siRNAs are produced at a low level from a variety of gene transcripts in the presence of Dicer2 in somatic tissues and, to a lesser degree, in the ovaries.

To directly attribute the enrichment of certain gene transcripts in the *Dcr2* mutant to regulation by endo-siRNAs, we compared the TPM ratios between antisense-mapping 21nt RNAs and RNA-seq transcripts in the wild-type mosquitoes for genes enriched, depleted, and non-differentially expressed in the *Dcr2* mutant. Genes that were enriched in the mutant line did not produce the most abundant antisense-mapping 21nt reads in the wild-type line (Fig. 5D). Furthermore, we identified genes with antisense-mapping 21nt reads present in at least 2 out of 3 biological replicates of the control line but not in any replicate of the *Dcr2* mutant line. In total, we identified 263 such genes in the midgut and 30 such genes in the ovary at a threshold of 0.1 RPM (Additional file 16: Table S2). At a significance level of adjusted *p* < 0.05, in the midgut, 17 transcripts were enriched and 20 depleted, which was significantly different from the total distribution of differentially regulated genes (*p* = 0.028, Fisher’s exact test on a 3 × 2 contingency table), with a greater number of both enriched and depleted transcripts than expected (expected enriched: 10.6, expected depleted: 14.0). In the ovary, 6 transcripts were enriched and 5 were depleted, which was in line with the overall distribution of differentially regulated genes (*p* = 0.24). Performing GSEA on these gene sets by themselves did not show any differential regulation in either tissue (midgut NES = − 1.14, false discovery rate = 0.19; ovary NES = − 0.81, false discovery rate = 0.73). In summary, although some gene transcripts putatively acting as a source of endo-siRNAs are enriched in the *Dcr2* mutant, we could not find a correlation between siRNA biogenesis and transcript enrichment.

## Discussion

In the present study, we compared the transcriptomic and small RNA landscapes between wild-type and *Dcr2*-deficient *Ae. aegypti* and showed that the lack of a functional *Dcr2* allele has wide and multifaceted effects on gene and TE expression in both midguts and ovaries in this genetic background. Our results confirm previously published detection of TE-derived endo-siRNAs in *Ae. aegypti* [38, 39], but their function as regulators of TE transcript abundance had not been studied until now. Despite a reduction of putative siRNAs originating from TEs in the *Dcr2* mutant (Fig. 3A), we did not observe a major enrichment of transcripts from autonomous TEs. Our data supports a family-specific yet collectively dispensable role of endo-siRNAs in TE regulation in *Ae. aegypti*.

We report numerous significant changes in TE transcript abundance in both directions, suggesting highly varying effects of a dysfunctional siRNA pathway on the expression of specific TE families (Additional files 9 and 17: Fig. S9 and Fig. S15 A). We also observe a concomitant reduction of TE-derived 21nt RNAs, even though our miRNA-normalized RPM values for the ovary need to be considered with caution due to *Dcr1* transcript enrichment. Nonetheless, combining the RNA-seq and small RNA-seq datasets shows that the TE families that are more expressed in the *Dcr2* mutant are not the source of more abundant siRNAs in the wild-type control (Fig. 3B). Similarly, gene transcripts from which 21nt RNAs were generated under control conditions were not strongly affected by their absence in the *Dcr2* mutant. This suggests that the enrichment of these TE and gene transcripts is due to an indirect effect of the *Dcr2* mutation. Beyond its role in RNA interference, Dicer2 has been shown to have a role in immune gene expression, cytoplasmic polyadenylation, and heterochromatin stability in insects [13, 14, 40–42]. Still, it is possible that certain TE families are more sensitive to siRNA-mediated silencing and thus become enriched in the *Dcr2* mutant despite levels of siRNA biogenesis in the wild-type control being akin to those of less sensitive families. It must, however, be noted that transcript abundance is not necessarily equivalent to autonomous expression, as TEs are commonly found in chimeric transcripts and the expression of non-functional TE fragments could be driven by nearby genes, just as TEs themselves can act as *cis*-regulators [43–46]. Our RT activity assay addresses the issue of detecting dysfunctional transcripts in RNA-seq data to some extent through functional confirmation but yields no information regarding specific families. Elucidating whether differential expression of TE families arises from a side effect of the *Dcr2* mutation, differential sensitivity to siRNA-mediated silencing, or a by-product of differential gene expression would require further studies on the reliance of individual TE families on specific TE regulation mechanisms in *Ae. aegypti*. In their current form, our data support an auxiliary function of endo-siRNAs in *Ae. aegypti*, acting non-specifically and with little consequence in a steady-state physiological setting.

In the re-analyzed whole-mosquito RNA-seq data of an *Ago2* mutant from a different genetic background [31], we saw enrichment of multiple orders of TEs (Additional file 2: Fig. S2). However, we also observed marked reductions in the expression of piRNA- and histone modification-related genes (Additional file 15: Fig. S14 C). Furthermore, the original authors themselves report reduced histone abundance in their mutants, leading to defects in DNA repair [31]. Heterochromatin stability is essential to TE repression, and both siRNAs and piRNAs have been implicated in transcriptional silencing of TEs [47–49]. Given the multiple potential causes for increased TE expression, it becomes difficult to implicate the direct, canonical action of the endo-siRNA pathway in TE enrichment in this *Ago2* mutant.

We did not find evidence of compensation for the lack of siRNAs through increased piRNA ping-pong activity. In general, although piRNAs are readily detectable in the midgut of *Ae. aegypti*, ping-pong activity appears restricted to a handful of TE families. In the ovaries, we did not observe a change in ping-pong signature other than a weak downward trend in piRNA abundance (Additional files 4 and 5: Fig. S4 and Fig. S5). We noticed that several genes important for piRNA biogenesis were depleted, although the piRNA-related gene set as a whole was not significantly affected (Additional files 14 and 15: Fig. S13 and Fig. S14B). The increase in RT activity and upward trend of LINE expression seen in the ovaries could thus stem from changes in piRNA pathway activity. Recent studies suggest potential cross-talk between siRNAs and piRNAs in insects, with maternally inherited siRNAs initiating piRNA cluster formation in *D. melanogaster* [50], and viral piRNAs appearing after viral siRNAs in *Ae. aegypti* [39]*.* Interestingly, we also saw collective depletions of piRNA pathway genes in two of our re-analyzed datasets of alternative siRNA pathway mutants (Additional file 15: Fig. S14 C-D). This confounding effect of siRNA pathway dysfunction may partially obscure the direct effect of endo-siRNAs. Our data, however, cannot conclusively confirm the role of siRNAs in piRNA biogenesis.

Since both endo-siRNAs and piRNAs are produced in the same tissues, there is likely a degree of redundancy between the two systems [51]. In *D. melanogaster*, for example, both endo-siRNAs and piRNAs are involved in the maintenance and initiation of heterochromatin [48, 50, 52, 53]. Indeed, both small RNA pathways have been shown to have redundant functions in general suppression of TE expression in *D. melanogaster* somatic tissues [17]. The Piwi-mediated establishment of heterochromatin during embryogenesis [54], coupled with expression of piRNA pathway components in non-gonadal tissues [55, 56], appears largely sufficient to repress TE expression in the adult fly, and the siRNA pathway is capable of suppressing TEs in a *Piwi* mutant line [17]. When both *Dcr2* and *Piwi* are mutated, however, there is a dramatic impact on overall TE expression (Additional file 2: Fig. S2). Our re-analysis of RNA-seq data from *D. melanogaster Dcr2* mutants show that even though some families may be directly enriched in the absence of endo-siRNAs, other forms of TE regulation are largely sufficient for general TE repression. Indeed, the seminal findings by Chung et al. [20] and Ghildiyal et al. [22] show rather modest enrichment of TE transcripts in reverse transcription quantitative polymerase chain reaction (RT-qPCR) data from non-gonadal tissues with substantial variation by TE family. The fact that endo-siRNAs *can* regulate some TEs does not appear to imply that they always *do* regulate all TEs in these dipteran species. It would, nonetheless, be of further interest to investigate changes in heterochromatin structure in Dicer2-deficient mosquitoes, as well as potential small RNA-independent mechanisms of TE regulation, such as zinc-finger proteins [57].

It is possible that mosquitoes from this genetic background, which are viable and fertile despite a dysfunctional *Dcr2* gene [32], are even less reliant on the endo-siRNA pathway for TE regulation. The lack of *Dcr2* in this genetic background also had little impact on vector competence for arboviruses, indicating a less significant role for siRNAs in antiviral defense than previously thought [32]. We previously observed that this *Dcr2* mutant line exhibits a range of modest fitness defects, such as slower development, increased pupal mortality, smaller adult body size, and reduced adult survival [32]. It is possible that some of these fitness defects reflect the modified patterns of TE and gene expression, although additional investigation is required to support this hypothesis. Artificially stimulating retrotransposon activity was found to promote aging in *D. melanogaster* [58]. In other organisms, somatic TE mobilization is involved in various health defects such as cancer, aging, and neurodegenerative diseases [1].

We found that gene-derived endo-siRNAs are produced at low levels in *Ae. aegypti* midguts, and to a lesser extent in the ovaries. Although some genes were a source of putative endo-siRNAs, they were not overrepresented among the transcripts that were enriched in the *Dcr2* mutant. Overall, these findings suggest that endo-siRNAs in *Ae. aegypti* are generated in small amounts and have only a minor impact on gene regulation. Nevertheless, it is important to note the limitations of our analyses. Our approach focused on mapping antisense reads and may have overlooked endo-siRNAs originating from other sources, such as those derived from long hairpins and acting in *trans* to repress their target gene [21, 59–61]. The expression of the source gene would thus not necessarily be affected by the presence of such endo-siRNAs.

## Conclusions

Our data are consistent with an auxiliary role of endo-siRNAs in silencing TE expression in *Ae. aegypti*, with individual TE families in different tissues displaying differing responses to a dysfunctional siRNA pathway in this genetic background. The endo-siRNA pathway is thus neither a sole nor a key regulator of TE expression in *Ae. aegypti.*

## Methods

### Mosquitoes

The *Dcr2*^R172fsX^ mutant line was generated as previously described [32] by introducing a premature stop codon in the 5 th exon of the *Dcr2* gene using clustered regularly interspaced short palindromic repeats/CRISPR‐associated protein 9 (CRISPR/Cas9)-mediated gene editing in a mosquito strain originally from Gabon. The resulting *Dcr2*-encoded peptide is thus truncated at the 173rd of its 1678 residues, containing only its DEAD-box domain. A “sister” control line with the wild-type *Dcr2* gene in a shared genetic background was derived from the same crossing scheme [32]. Prior to the experiments, eggs were hatched in dechlorinated tap water and larvae were reared on a standard diet of Tetramin (Tetra) fish food as previously described [32]. After emergence, adults were maintained in insect cages (BugDorm) under a 12 h–12 h light–dark cycle with ad libitum access to a 10% sucrose solution.

### RNA extraction

Three biological replicates of 20 5- to 8-day-old females from both the *Dcr2* mutant and wild-type control lines each (16 th generation) were dissected into three parts—thorax, midgut, and ovaries—and snap frozen in dry ice. Total RNA was extracted from the pools with TRIzol (Life Technologies; ref. 15596026). The tissues were homogenized in 500 μl of TRIzol reagent using a Precellys homogenizer (Bertin Technologies). The RNA was phase-separated using chloroform, bound by linear acrylamide (Invitrogen; ref. AM9520) and precipitated in isopropanol. After two washes with 70% ethanol, the RNA was dissolved in 40 μl of RNase-free water. RNA concentration and purity was verified using NanoDrop (ThermoFisher Scientific). Due to excessive degradation seen in the small RNA sequencing data for thorax samples (Additional file 18: Fig. S16), these were later excluded from the analysis.

### RNA sequencing

One to two micrograms of total RNA per sample were treated with DNase I (Invitrogen; AM2224), of which 200–500 ng were used for RNA-seq. RNA quality was verified using Agilent 2100 Bioanalyzer. The RNA-seq library was prepared with the Illumina Stranded mRNA Prep kit. Paired-end sequencing (2 × 150 cycles) was performed with a depth of 30 million paired-end reads on an Illumina NovaSeqX platform. Read quality was assessed using fastQC v0.11.9 [62] and MultiQC v1.12 [63]. Raw RNA-seq reads are available from the Gene Expression Omnibus (GEO) archive under accession number GSE275899 [64].

### RNA-seq data analysis

RNA-seq data were analyzed using the rnaflow pipeline [65]. In brief, raw reads were trimmed using cutadapt v2.10 [66] using the parameters “-m 25 -O 6 --trim-n --max-n 1 -q 30.” Reads were aligned to the AaegL5 *Ae. aegypti* reference genome (VectorBase release 61) [67] using STAR v2.7.9a [68] two-pass alignment with the options “--outFilterMismatchNoverLmax 0.05 --outFilterMultimapNmax 50.” Reads were counted using TEtranscripts v2.2.3 [69] using “--mode multi.” A principal component analysis (PCA) of variance-stabilized counts was done using the R package FactoMineR v2.9 [70]. Differential expression analysis was performed on a concatenated table of gene and TE counts using DESeq2 v1.40.2 [71] for each tissue separately. Genes with a total raw read count < 10 across all replicates and both conditions within a given tissue were excluded from the analysis. Detection of chimeric reads was done using ChimeraTE v1.1.1 [72]. For transcripts per million (TPM) values, the transcript length used for count normalization was defined as the total, non-overlapping length of all exons (“collapsed” exons) of each gene. For TEs, transcript length was defined as the length of the family consensus. Gene set enrichment analysis was performed on the DESeq2 output using the R package fgsea v1.26.0 [73] on genes and TEs separately. For TEs, individual families were grouped by their order. Genes were grouped according to Kyoto Encyclopedia of Genes and Genomes (KEGG) [74] (release 106) pathway annotations. Three additional gene sets were added, containing either siRNA-, piRNA-, or histone modification-related genes based on homology to selected *D. melanogaster* gene ontology (GO) terms (Additional file 19: Table S3). Homology was determined using annotation from VectorBase release 66. Ensembl gene IDs were transformed into AAEL nomenclature using the online tool DAVID [75]. All plots were made using the R package ggplot2 v3.4.4 [76]. The threshold for statistical significance for this and all other analyses was set to *p* < 0.05.

### RNA-seq data re-analysis

Published RNA-seq datasets of an *Ago2* mutant in *Ae. aegypti* [31] and of two *Dcr2* mutants in *D. melanogaster* [17, 37] were re-analyzed following the same pipeline as described above. Read archive accession numbers of the re-analyzed datasets are specified in Additional file 20: Table S4. For data from *D. melanogaster*, the TE annotation was taken from Daron et al. [36]. The genomic sequence, gene annotation, and TE consensus sequences were retrieved from FlyBase [77] release 2023_02. The KEGG pathway annotation was retrieved from the KEGG database (release 113). For data from Beek et al. [17], where both single-end and paired-end sequencing strategies were used, reads were aligned separately and the counts later concatenated for both single- and paired-end datasets.

### Small RNA sequencing and data pre-processing

Five micrograms of total RNA per sample was used directly for small RNA sequencing. Small RNAs of 19–33 nt in length were purified from 15% acrylamide/bisacrylamide (37.5:1), 7 M urea gel as previously described [78]. The small RNA library was prepared using NEB Next Multiplex Small RNA Library Prep kit for Illumina (New England Biolabs [NEB]; Ipswitch, MA, USA; ref. E7300 L) with Universal miRNA Cloning Linker (NEB; ref ES1315S) as the 3’ adaptor and in-house designed indexed primers (Additional file 21: Table S5). Libraries were diluted to 4 nM and sequenced using an Illumina NextSeq500 High Output kit v2 (75 cycles) on an Illumina NextSeq500 platform over 52 cycles. Raw reads were pre-processed through poly-A/T/C/G removal (trimming 10 or more consecutive identical bases) and adapter trimming using cutadapt v2.10 [66] with options “-e 0.15 -O 6 --trimmed-only -m 18 --match-read-wildcards -q 20.” Raw small RNA-seq reads trimmed for adapters, low-quality sequences, and poly-N are available from the GEO archive under accession number GSE275903 [79].

### TE-derived 21nt RNA analysis

Pre-processed reads (i.e., reads trimmed for adapters, low-quality sequences, and poly-N) were aligned to the AaegL5 *Ae. aegypti* reference genome (VectorBase release 61) using bowtie v1.2.3 [80] using the options “-v 1 -a -M 1” (random attribution of multimapping reads and allowing one mismatch). Reads that mapped to annotated micro-RNAs (miRNAs), transfer RNAs (tRNAs), small nuclear RNAs (snRNAs), and small nucleolar RNAs (snoRNAs), and an unannotated miRNA, aae-miR-989 [81], and a gene (*AAEL019428*) containing a highly expressed aae-miR-2942 precursor, previously detected [82] in *Ae. albopictus* as aal-miR-956p with one mismatch, were filtered out using BEDTools intersect [83] and Picard FilterSamReads [84], requiring the full read to be within the annotated gene (Additional file 22: Table S6). To filter out 21nt piRNA fragments from the alignment, pre-processed reads were collapsed and dusted using the small RNA NGS toolbox v2.1 [85], mapped to the reference genome using sRNAmapper v1.0.5 [85] (option “-alignments best”) and used to annotate piRNA clusters using proTRAC v2.3.1 [86] with default options. piRNA clusters were annotated for each sample and merged across the three biological replicates for each tissue and condition (*Dcr2* mutant or wild-type) pair (Additional file 23: Table S7). 21nt reads mapping to annotated clusters were filtered out from the alignment. Finally, 21nt RNAs were counted using TEtranscripts v2.2.3 (option “--mode multi”). Reads per million mapped reads (RPM) values for each order of TEs were normalized by the total miRNA RPM, under the assumption that the size of the total miRNA pool remains constant within a given tissue across replicates and conditions. For TPM values, the read counts were normalized against the length of collapsed exons for genes and the family consensus length for TEs and subsequently normalized by the total miRNA TPM. Additionally, whole-mosquito small RNA-seq data from an independent *Dcr2* mutant line of *Ae. aegypti* [33] (Additional file 20: Table S4) were re-analyzed through the same pipeline and the same piRNA cluster coordinates (merged across all tissues).

### Gene-derived 21nt RNA analysis

Potential gene sources of endo-siRNAs were identified using a similar pipeline as above with minor modifications. First, only uniquely mapping reads were considered (bowtie options “-v 1 -a -m 1”) and all tRNA-, miRNA-, snRNA-, snoRNA-, and piRNA cluster-mapping reads were filtered out as described above. Second, to circumvent contamination by fragments of transcripts, only the antisense-mapping reads were counted (TEtranscripts option “--stranded reverse”). Genes potentially acting as a source of endo-siRNAs were defined as genes with more than 0.1 RPM of antisense-mapping reads (rounded to the nearest whole number of counts) prior to normalization by the total miRNA RPM. To calculate the RPM and TPM values, the whole of mapped reads was used as the denominator, i.e., including non-uniquely mapping reads and reads mapping in both orientations. The number of genes acting as a source of siRNAs in the two mosquito lines was compared through a negative binomial generalized linear model using the R package MASS v60.0.1 [87]. Antisense mapping reads for analysis of size distribution were extracted directly from the alignment files by intersecting the alignments with collapsed exons using BEDTools intersect (options “-f 0.5 –S”).

### Ping-pong signature analysis

The size range of potential piRNAs was defined to 26–30 nt based on read size distribution (Additional file 17: Fig. S15). For piRNA analysis, pre-processed reads (i.e., reads trimmed for adapters, low-quality sequences, and poly-N) were aligned using bowtie v1.2.3 with the options “-v 1 -a --best --strata” and filtered for miRNA, tRNA, snRNA, and snoRNA genes as described above. The alignments were then subset by TE order and strand with relation to the annotated TE copy and filtered for reads mapping to more than one orientation relative to all copies within the order or more than one order of TEs. Reads mapping to the antisense strand were considered potential primary piRNAs and intersected with read mapping to the sense strand, considered potential secondary piRNAs. Overlapping pairs where the potential primary piRNA was upstream of the potential secondary piRNA on the sense strand of the TE were counted and normalized by the number of mappings of each member of the pair, e.g., a pair of 2 reads mapping to 10 places each and overlapping each other in 5 of these places would contribute 0.25 overlapping pairs. Logo plots of potential and putative ping-pong (i.e., overlapping by 10 nt) primary and secondary piRNAs were generated using the plot_seqlogo functionality of the biopieces package [88]. Counts for sense, antisense, and putative primary and secondary reads were generated by extracting unique read names from the subset alignments. Further investigation of putative secondary piRNAs was conducted by separating secondary piRNA reads based on which set of copies they map to in order to identify the sources of ping-pong signatures.

### Reverse transcriptase activity assay

Reverse transcriptase (RT) activity was measured in midguts and ovaries using a protocol adapted from Wu et al. [89] and Pyra, Böni and Schüpbach [90]. Four pools of 5 tissues from the *Dcr2* mutant line and the wild-type control line (24 th generation) were collected at 5–7 days post adult emergence, with midguts and ovaries originating from the same mosquitoes. Organs were dissected and placed in 100 μL of 3-[(3-cholamidopropyl)dimethylammonio]−1-propanesulfonate (CHAPS) lysis buffer [91], containing 10 mM Tris–HCl pH 7.5 (Invitrogen; ref. AM9855G), 400 mM NaCl (Invitrogen, ref. AM9759), 1 mM MgCl_2_ (Invitrogen; ref. AM9530G), 1 mM EGTA (Sigma-Aldrich; ref. E4378), 0.5% CHAPS (ThermoFisher Scientific; ref. 28300), 10% glycerol (Sigma-Aldrich; ref. J61059-AP), 1 mM dithiothreitol (DTT) (Invitrogen; ref. Y00147), and 1 × cOmplete EDTA-free protease inhibitor cocktail (PIC) (Roche; ref. 11873580001). The tissues were homogenized in a Precellys homogenizer (Bertin technologies) and stored at − 80 °C. Subsequently, the samples were clarified by centrifugation for 10 min at 16,000* g* to remove tissue debris. The resulting supernatant was collected into a new tube. Protein content in the supernatant was measured using Pierce Detergent Compatible Bradford Assay kit (ThermoFisher Scientific; ref. 23246) according to the manufacturer’s instructions. All samples were diluted to the protein concentration of the least concentrated sample (71 ng/μL). Prior to the RT reaction, 30 pmol of MS2 reverse primer (5′CGCTTGTAGGCACCTTGATC) with 0.4 mM dNTPs (ThermoFisher Scientific; ref. R1121) was allowed to anneal to 100 ng of MS2 RNA (Roche; ref. 10165948001) in a 12.5-μL annealing reaction at 65 °C for 5 min. The annealing reactions were used in subsequent 25-μL RT reactions, containing 30 pmol MS2 reverse primer, 100 ng MS2 RNA, 0.2 mM dNTPs, 1 mM DTT (Invitrogen; ref. Y00147), 10 mM Tris–HCl pH 7.5 (Invitrogen; ref. AM9855G), 1 mM KCl (Invitrogen; ref. AM9640G), 0.14 mM MnCl_2_ (Sigma-Aldrich; ref. M1787), 0.02% Triton X-100 (Sigma-Aldrich; ref. 93433), 40 units of RNase OUT (Invitrogen; ref. 10777–019), and 5 μL of protein sample. The RT reaction was allowed to progress for 1 h at 25 °C, after which it was stopped by heat inactivation at 70 °C for 15 min. Heat-inactivated samples (45 min of incubation at 98 °C), CHAPS buffer alone, and no-template reactions served as negative controls. Three technical replicates of 1 unit of SuperScript II (Invitrogen; ref. 18064022) were used for relative quantification of RT activity. Generated MS2 cDNA was quantified with two technical replicates through qPCR in a 10-μL reaction containing 3 pmol MS2 forward primer (5′TCCCGGTCGTTTTAACTCGA), 3 pmol MS2 reverse primer (5′CGCTTGTAGGCACCTTGATC), and 1 μL of RT reaction (template) using Promega GoTaq qPCR Master Mix (Promega; ref. A600 A). Statistical analysis was done using a linear mixed effect model using the R packages lme4 v1.1–35.1 [92] and lmerTest v3.1–3 [93] with organ and condition as explanatory variables and biological replicate within each condition as a blocking variable (degrees of freedom estimation method: Kenward-Roger). *P*-values for pairwise comparisons were calculated using Wald’s test through the package emmeans v1.9.0 [94].

## Supplementary Information

Additional file 1: Figure S1 – MITEs and UDs are often found in chimeric transcripts. Proportion of TE families in each order implicated in chimeric transcripts for midgut and ovary RNA-seq data. Matrix of regression coefficients for midgut and ovary RNA-seq data. The parameters base. Mean, the log_2_ fold-change in the *Dcr2* mutant, a combined metric [99] for base expression and log_2_ fold-change, and the DESeq2 statistic for genes implicated in chimeric transcripts were regressed as a function of the same parameter for implicated TEs. A thin circle in the center of each grid intersection denotes the limit for statistical significance of the slope coefficient for the regression. Negative-log_10_-transformed adjusted *p*-values > 20 are denoted with a thick outer circle. Significant positive coefficients for comparisons of all parameters are seen for LTR, MITE, and undetermined transposons in the midgut. Since most of MITE and UD families are also implicated in gene-TE chimeric transcripts, some of the expression of these orders may be attributed to the expression of adjacent genes. For LTR transposons, positive regression coefficients are seen both in the midgut and the ovaries. However, the fraction of LTR transposons implicated in chimeric transcripts is much smaller.

Additional file 2: Figure S2 – Re-analysis of TE expression in published RNA-seq data from *Ae. aegypti* and *D. melanogaster* mutants. The top two rows show MA plots and gene set enrichment analysis results from our study for all orders of TEs. Subsequent rows show MA plots and GSEA results from the re-analysis of publicly available RNA-seq data for the *Ae. aegypti Ago2* mutant and *D. melanogaster Dcr2* mutants or a double *Dcr2* + *Piwi* knockout mutant, bottom row. In the MA plots, the x-axis shows mean read counts normalized by the median of ratios and the y-axis shows log_2_ fold-change in the mutant line. TE families are colored according to their differential expression. The dotted line in the center of each plot represents the mean log_2_ fold-change. In the GSEA results, the height of each bar represents the normalized enrichment score, i.e., the relative, rank-based enrichment of the TE order compared to a random group of transcripts with the same size. Numbers above or below the bars indicate the false discovery rate for the enrichment or depletion in the mutant relative to the wild-type control.

Additional file 3: Figure S3 – TE-mapping 21nt RNAs are reduced in an independent *Dcr2* mutant line. Re-analysis of small RNA-seq data from whole mosquitoes of the *Dcr2* mutant in Samuel et al*.*, [33]. The four bar plots show the four sets of mosquitoes infected with dengue virus 2 (DENV2, top left), dengue virus 4 (DENV4, top right), Sindbis virus (SINV, bottom left), and yellow fever virus (YFV, bottom right). The y-axis values show the RPM values for 21nt reads mapping to the TE orders specified on the x-axis. The RPM values were normalized by the RPM of the total miRNA pools, assuming that the total amount of miRNAs does not change between conditions and viral infections. The error bars denote one standard deviation. Significant differences determined by Welch’s t-test are indicated by asterisks: *p < 0.05, **p < 0.01.

Additional file 4: Figure S4 – Sense and secondary piRNA abundance is equivalent between the *Dcr2* mutant and control lines. (A) Amounts of putative secondary piRNAs for each TE order for midguts (top) and ovaries (bottom). The error bars denote one standard deviation. The numbers above the bar plots indicate *p*-values obtained with Welch’s t-test. For SINE, no secondary piRNAs were detected in the midgut samples. (B) Amounts of sense piRNA-sized reads for each TE order in midguts (top) and ovaries (bottom). The fraction of these that are considered putative secondary piRNAs is highlighted in dark. The error bars denote one standard deviation. Grey numbers above the bars indicate the *p*-values obtained with Welch’s t-test for the amount of sense piRNA-sized reads. Black numbers indicate *p*-values for putative secondary piRNAs.

Additional file 5: Figure S5 – Antisense and ‘ping-pong’-interacting primary piRNA abundance is equivalent between the two mosquito lines. (A) Amounts of putative primary piRNAs identified by 10nt overlap-based analysis of ping-pong signature (PP-primary) for each TE order for midgut (top) and ovary (bottom) samples. The error bars denote one standard deviation. The numbers above the bar plots indicate *p*-values obtained with Welch’s t-test. For SINE, no 10nt overlaps and thus no ‘ping-pong’-interacting primary piRNAs were detected in the midgut samples. (B) Amounts of antisense piRNA-sized reads for each TE order in midgut and ovary samples. The fraction of these that are considered putative primary piRNAs identified through 10nt overlap-based analysis of ping-pong signature (PP-primary) is highlighted in dark. The error bars denote one standard deviation. Grey numbers above the bars indicate the *p*-values obtained with Welch’s t-test for the amount of antisense piRNA-sized reads. Black numbers indicate *p*-values for putative ‘ping-pong’-interacting primary piRNAs

Additional file 6: Figure S6 – Midgut piRNA-sized reads overlapping each other by 10 nt originating from LTR transposons are dominated by a single copy in wild-type mosquitoes. (A) The genomic origin of reads involved in 10nt overlaps in the midgut. For each TE order and replicate pool, the proportion of 10nt overlaps originating from a given set of TE copies is shown. The letter index following the family name for each set of copies is arbitrary. For each order, up to 20 sets of copies ordered by their contribution to total 10nt overlaps, or those necessary to reach 90% of total 10nt overlaps across all samples, were assigned colors. All other copy sets were assigned black and grouped into ‘Others’. (B) The composition of each set shown in (A), illustrating the size of the set as well as shared and unique copies between sets. Individual copies present in any of the sets for a given order are alphabetically ordered by genomic position on the x-axis. Copies present in each set on the y-axis are marked by a black bar. The names of the sets on the y-axis follow the same order and coloring scheme as in the legend of (A)

Additional file 7: Figure S7 – Excluding the single locus contributing to the vast majority of 10nt overlaps for LTR transposons in the midgut abolishes the only difference between the *Dcr2* mutant and control lines. (A) Box plots of 10nt overlap Z-scores among 26-30nt sense and antisense reads mapping to TEs in midguts (top) and ovaries (bottom). *P*-values indicated above the box pairs were obtained using Welch’s t-test. (B) Frequency of overlaps among sense and antisense reads by a given number of nt for midguts (top two rows) and ovaries (bottom two rows). Reads mapping to the copy of TE_0669_Gypsy that dominated the overlaps in control mosquito midguts were excluded from the plot for LTR transposons for the midgut samples.

Additional file 8: Figure S8 – No prominent 10 A bias for non-autonomous TE orders. Logo plots were constructed from 26-30nt reads mapping to the sense and antisense strands of TEs. Each column of logo plots in the figure corresponds to a TE order (MITE, SINE, UD = undetermined). The top half of the figure shows logo plots for the midgut samples, while the bottom half shows logo plots for the ovary samples. Within each half, the top half corresponds to samples from the *Dcr2* mutant line, while the bottom corresponds to samples from control mosquitoes. Within each condition-organ-order partition, the three sets of four logo plots correspond to the three biological replicates. Each set is composed of four logo plots in the order top to bottom: 1) reads mapping to sense strand; 2) putative secondary piRNAs, i.e., reads mapping to sense strand and overlapping a putative primary piRNA in the secondary position (downstream of the corresponding putative primary piRNA) by 10 nt; 3) putative primary piRNAs engaged in the ‘ping-pong’ cycle, i.e., reads mapping to the antisense strand and overlapping a putative secondary piRNA in the primary position (upstream of the corresponding secondary piRNA) by 10 nt; 4) reads mapping to the antisense strand. The number of reads used to construct the logo is specified on the right side in each plot.

Additional file 9: Figure S9 – Volcano plots and summary bar plots for differentially expressed genes and TE families show a wide perturbation of expression. Plots to the left of the partition correspond to genes, while plots to the right of the partition correspond to TEs. The three rows of plots correspond to the organs (ordered top to bottom): thorax, midgut, and ovary. The bar plots to the right of the volcano plot summarize the number of depleted (DOWN), non-differentially expressed (NO), and enriched (UP) genes or TEs in the *Dcr2* mutant line relative to the control line. Genes or TE families with an absolute log_2_ fold-change > 1 and an adjusted *p*-value < 0.05 are colored according to the direction of their differential expression (red: enriched; blue: depleted; grey: not differentially expressed).

Additional file 10: Figure S10 – Correlation between TE-derived 21nt RNAs and differential TE expression for all orders. (A) miRNA-adjusted reads per million mapped reads (RPM) mapping to the different TE orders in the midgut (left plot) and ovaries (right plot) of *Dcr2* mutant and control mosquitoes. Error bars denote one standard deviation. Statistical significance was determined using Welch’s t-test (*p < 0.05, **p < 0.01, NS = non-significant). (B) Ratios between miRNA-adjusted 21nt RNAs expressed in transcripts per million (TPM) and RNA-seq TPM in the control mosquitoes for individual TE families depleted (Down), non-differentially expressed (Not DE), and enriched (Up) in the *Dcr2* mutant within each TE order for midguts (top two rows) and ovaries (bottom two rows). Statistical significance was determined using Wilcoxon rank sum test (*p < 0.05, **p < 0.01, ***p < 0.001, NS = non-significant). Families with no detected transcripts were excluded. Families with detected transcripts but no detected 21nt RNAs are shown below the dashed line.

Additional file 11: Table S1 – Differential expression matrix of genes and TE families. Output of DESeq2 for all (original and re-analyzed) RNA-seq datasets ordered by statistical significance (adjusted p-value). Raw counts are also supplied in separate sheets

Additional file 12: Figure S11 – Logo plots show 1U and 10 A bias in putative piRNAs only for certain autonomous TE orders. Logo plots were constructed from 26-30nt reads mapping to the sense and antisense strands of TEs. Each column of logo plots in the figure corresponds to a TE order (DNA, LINE, LTR). The plot is structured as in Additional file 8: Fig. S8

Additional file 13: Figure S12 – Sequence composition of secondary piRNAs originating from the single locus mainly contributing to the 10nt overlaps among LTR transposons in the midgut of control mosquitoes. Logo plots derived from sequences mapping to the set of copies TE_0669_Gypsy_BTN, consisting of one single copy as seen in Additional file 6: Fig. S6B. Top half of figure shows logo plots from the three replicates of the *Dcr2* mutant mosquitoes and bottom half shows the three replicates of the control mosquitoes.

Additional file 14: Figure S13 – Gene set enrichment analysis for genes shows multiple differentially regulated pathways in the ovaries. (A) GSEA for annotated KEGG pathways as well as manually added pathways ‘siRNA’, ‘piRNA’, and ‘Histone Modification’ for midgut (left) and ovary (right) samples. Significant enrichments or depletions are labelled with asterisks indicating the false discovery rate (*p < 0.05, **p < 0.01, ***p < 0.001). (B) Differential expression of *Ae. aegypti* homologs of *D. melanogaster* genes associated with the siRNA-, piRNA-, and histone modification-related pathways. Gene names in bold font indicate either a unique homolog, or a homolog with an annotated homologous function. Within each colored circle, a thin black circle indicates the threshold for statistical significance (*p* = 0.05). The size of the colored circle corresponds to the significance level for differential expression expressed as –log_10_(*p*-value). Significantly differentially expressed genes are highlighted with an asterisk in the plot. Genes with a negative-log_10_-transformed *p*-value > 20 have their colored circles surrounded by a thick outer circle. A horizontal line separates the genes with any significant differential expression from those with none.

Additional file 15: Figure S14 – Gene set enrichment analysis shows several cases of depleted pathways involved in TE regulation among all siRNA-pathway mutant datasets. The results of GSEAs for the the gene sets of interest with siRNA-, piRNA-, and histone modification-related (HistMod) functions for *Ae. aegypti* midguts (our data – A), ovaries (our data – B), and whole mosquitoes (Dong & Dimopoulos, [31] – C), and *D. melanogaster* carcasses (Roy et al. [37] – D), ovaries (Roy et al. [37] – E), and heads (Beek et al. [17] – F). The height of each bar represents the normalized enrichment score (NES), i.e., the relative, rank-based enrichment of the gene set compared to a random group of transcripts with the same size. Numbers above or below the bars indicate the false discovery rate for the enrichment (red bars) or depletion (blue bars) in the mutant relative to the wild-type control. Significant enrichments or depletions are highlighted in bold and with asterisks (*p < 0.05, **p < 0.01, ***p < 0.001).

Additional file 16: Table S2 – Genes implicated in endo-siRNA biogenesis. All genes surpassing the whole number 0.1 reads per million mapped reads (RPM) equivalent number of counts in the antisense orientation in midgut and ovary samples. Genes surpassing the threshold in at least 2 out of 3 control (Sis) replicates and none of the *Dcr2*^R172fsX^ (Dcr) replicates are included in a separate sheet, as well as their differential expression.

Additional file 17: Figure S15 – Size distributions of small RNA reads following different levels of filtering. The plots are structured in three main columns – thorax samples are on the left, midgut samples in the center, and ovary samples on the right. Within each partitioned region, control mosquito libraries are shown on the left and *Dcr2* mutant libraries are shown on the right. The rows correspond to reads subset based on filtering: First row – adapter-trimmed only (‘unfiltered/raw’), second row – filtered for reads mapping to miRNA, tRNA, snRNA, and snoRNA genes, third row – further filtered for reads mapping to annotated piRNA clusters, fourth row – only reads that map to piRNA clusters but not to any miRNA, tRNA, snRNA, or snoRNA genes. The percentage of 21nt reads is added to each plot. The error bars denote one standard deviation. When filtered for small RNA genes, a ‘block’ of reads can be seen in thorax samples, attributable to RNA degradation, while a piRNA-sized ‘hump’ can be seen most clearly in ovary samples, but also in midgut samples. Unfiltered reads from somatic tissues display a clear domination of the library by miRNA-sized reads, which are filtered away following intersection with annotated small RNA genes.

Additional file 18: Figure S16 – Size distributions of small RNA reads mapping to exons, TEs, and whole genes. Size distributions of small RNA-seq reads filtered for miRNA, tRNA, snRNA, and snoRNA genes mapping to exons (top row), TEs (center row), and whole genes (exons and introns, bottom row) in the three different tissues – midgut (left column), ovary (center column), and thorax (right column). Reads mapping to the sense strand are shown with a positive RPM, reads mapping to the antisense strand are shown with a negative RPM. Thorax samples in both the *Dcr2* mutant and the control mosquitoes show an overabundance of reads of various sizes mapping to the sense strand of genes and, in particular, the sense strand of exons.

Additional file 19: Table S3 – Gene sets of interest. Groupings of genes into gene sets, as well as the retrieved genes and their *Ae. aegypti* homologs. Retrieved KEGG pathways and their component genes are also included.

Additional file 20: Table S4 – Accession numbers and rudimentary metadata for re-analyzed datasets. NCBI [31, 33, 37] and EBI [17] accession numbers, sample names, and sequencing strategies for the re-analyzed data from *Ae. aegypti* and *D. melanogaster Dcr2* or *D. melanogaster* double *Dcr2* + *Piwi* mutants.

Additional file 21: Table S5 – Primer sequences used for small RNA library preparation. Adapters and primers used in library preparation. Samples were indexed on the 3’ end during PCR amplification. Indexed primers are indicated with their associated samples. Control and *Dcr2*^R172fsX^ mosquitoes were sequenced on two separate flow cells and thus share the same indexes for tissues and replicates.

Additional file 22: Table S6 – BED file of small RNA genes. Coordinates used for filtering out small RNAs originating from small RNA genes. Reads mapping to coordinates labelled as ‘miRNA’ in the last column were used for scaling of RPM between samples

Additional file 23: Table S7 – piRNA clusters annotated by proTRAC. piRNA clusters for midgut, ovary, and thorax samples for both *Dcr2* mutant and control mosquitoes used for filtering piRNA fragments. The clusters were annotated individually for each biological replicate and subsequently merged (merging analogous to a full join) for each tissue-condition pair. Length, strand, and read density (reads per kilobase) are specified, when available, for all merged clusters, but the order of one set of a parameter’s values does not necessarily correspond to the order in the other parameters’ values for the same cluster.

## Data Availability

All relevant data are included in the main body of the article and in the Additional files. Raw RNA-seq reads and small RNA-seq reads are available from the GEO archive under accession numbers GSE275899 (ref. [64]) and GSE275903 (ref. [79]), respectively. The re-analyzed RNA-seq dataset for *D. melanogaster* *Dcr2* mutant ovaries and carcasses are available from the NCBI archive under the accession number PRJNA540249 (ref. [95]). The re-analyzed RNA-seq dataset for *D. melanogaster* *Dcr2* and *Piwi* mutant heads is available from the EBI archive under the accession number PRJEB25033 (ref. [96]). The re-analyzed RNA-seq dataset for the *Ae. aegypti **Ago2 *mutant is available from the NCBI archive under accession number PRJNA889408 (ref. [97]). The re-analyzed small RNA-seq dataset for the *Ae. aegypti* *Dcr2* mutant is available from the NCBI archive under accession number PRJNA691676 (ref. [98]).

## Abbreviations

Ago2: Argonaute 2
CHAPS: 3-[(3-Cholamidopropyl)dimethylammonio]-1-propanesulfonate
CRISPR/Cas9: Clustered regularly interspaced short palindromic repeats / CRISPR‐associated protein 9
Dcr2: Dicer2
dsRNA: Double-stranded RNA
DTT: Dithiothreitol
GSEA: Gene set enrichment analysis
KEGG: Kyoto Encyclopedia of Genes and Genomes
LINE: Long interspersed nuclear element
LTR: Long terminal repeat
miRNA: MicroRNA
MITE: Miniature inverted repeat transposable element
NES: Normalized enrichment score
nt: Nucleotide
PCA: Principal component analysis
PCR: Polymerase chain reaction
piRNA: Piwi-interacting RNA
qPCR: Quantitative PCR
RISC: RNA-induced silencing complex
RNA-seq: RNA sequencing
RPM: Reads per million mapped reads
RT: Reverse transcriptase
RT-qPCR: Reverse transcription quantitative PCR
siRNA: Small interfering RNA
snRNA: Small nuclear RNA
snoRNA: Small nucleolar RNA
SINE: Short interspersed nuclear element
TE: Transposable element
TIR: Terminal inverted repeat
TPM: Transcripts per million
