# Supplementary figures and images for: Limited impact of the siRNA pathway on transposable element expression in Aedes aegypti
Source: BMC Biol. 2025 May 13;23:130. doi: 10.1186/s12915-025-02225-8 (PMC12076837; doi:10.1186/s12915-025-02225-8)

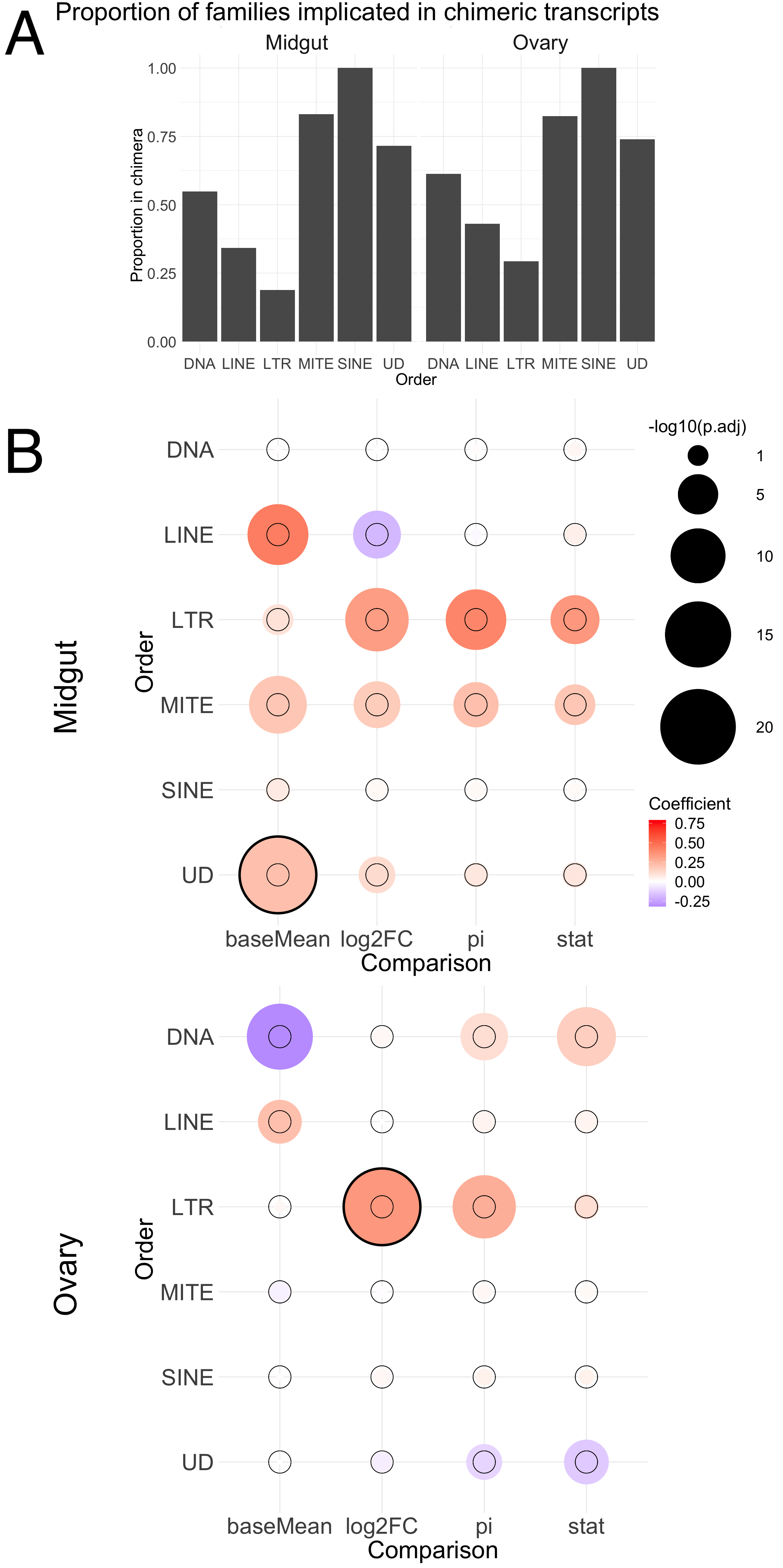

Supplement: Supplementary file 1 — Additional file 1: Figure S1 – MITEs and UDs are often found in chimeric transcripts. Proportion of TE families in each order implicated in chimeric transcripts for midgut and ovary RNA-seq data. Matrix of regression coefficients for midgut and ovary RNA-seq data. The parameters base. Mean, the log2 fold-change in the Dcr2 mutant, a combined metric [99] for base expression and log2 fold-change, and the DESeq2 statistic for genes implicated in chimeric transcripts were regressed as a function of the same parameter for implicated TEs. A thin circle in the center of each grid intersection denotes the limit for statistical significance of the slope coefficient for the regression. Negative-log10-transformed adjusted p-values > 20 are denoted with a thick outer circle. Significant positive coefficients for comparisons of all parameters are seen for LTR, MITE, and undetermined transposons in the midgut. Since most of MITE and UD families are also implicated in gene-TE chimeric transcripts, some of the expression of these orders may be attributed to the expression of adjacent genes. For LTR transposons, positive regression coefficients are seen both in the midgut and the ovaries. However, the fraction of LTR transposons implicated in chimeric transcripts is much smaller. [file 12915_2025_2225_MOESM1_ESM.png]

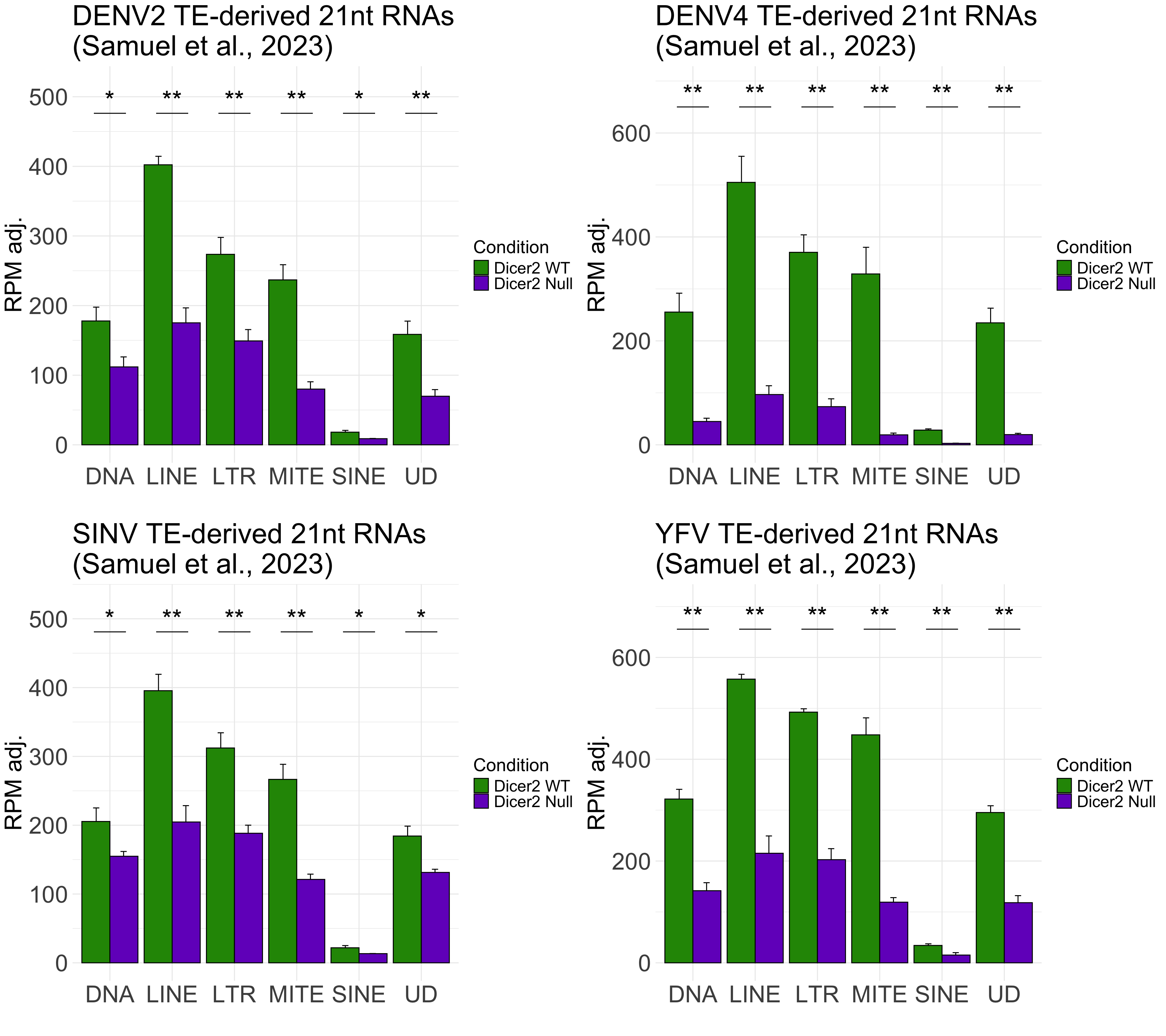

Supplement: Supplementary file 3 — Additional file 3: Figure S3 – TE-mapping 21nt RNAs are reduced in an independent Dcr2 mutant line. Re-analysis of small RNA-seq data from whole mosquitoes of the Dcr2 mutant in Samuel et al., [33]. The four bar plots show the four sets of mosquitoes infected with dengue virus 2 (DENV2, top left), dengue virus 4 (DENV4, top right), Sindbis virus (SINV, bottom left), and yellow fever virus (YFV, bottom right). The y-axis values show the RPM values for 21nt reads mapping to the TE orders specified on the x-axis. The RPM values were normalized by the RPM of the total miRNA pools, assuming that the total amount of miRNAs does not change between conditions and viral infections. The error bars denote one standard deviation. Significant differences determined by Welch’s t-test are indicated by asterisks: *p < 0.05, **p < 0.01. [file 12915_2025_2225_MOESM3_ESM.png]

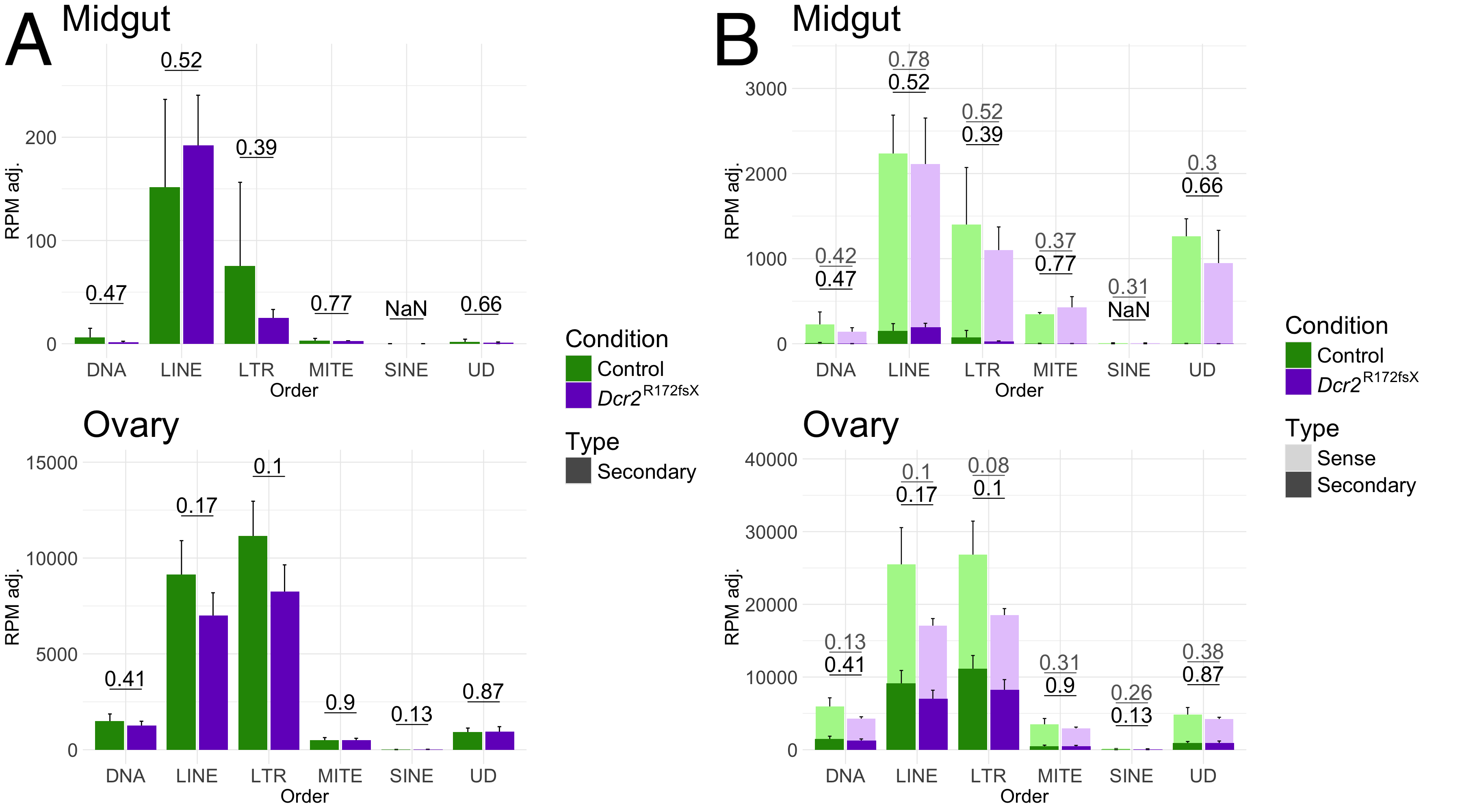

Supplement: Supplementary file 4 — Additional file 4: Figure S4 – Sense and secondary piRNA abundance is equivalent between the Dcr2 mutant and control lines. (A) Amounts of putative secondary piRNAs for each TE order for midguts (top) and ovaries (bottom). The error bars denote one standard deviation. The numbers above the bar plots indicate p-values obtained with Welch’s t-test. For SINE, no secondary piRNAs were detected in the midgut samples. (B) Amounts of sense piRNA-sized reads for each TE order in midguts (top) and ovaries (bottom). The fraction of these that are considered putative secondary piRNAs is highlighted in dark. The error bars denote one standard deviation. Grey numbers above the bars indicate the p-values obtained with Welch’s t-test for the amount of sense piRNA-sized reads. Black numbers indicate p-values for putative secondary piRNAs. [file 12915_2025_2225_MOESM4_ESM.png]

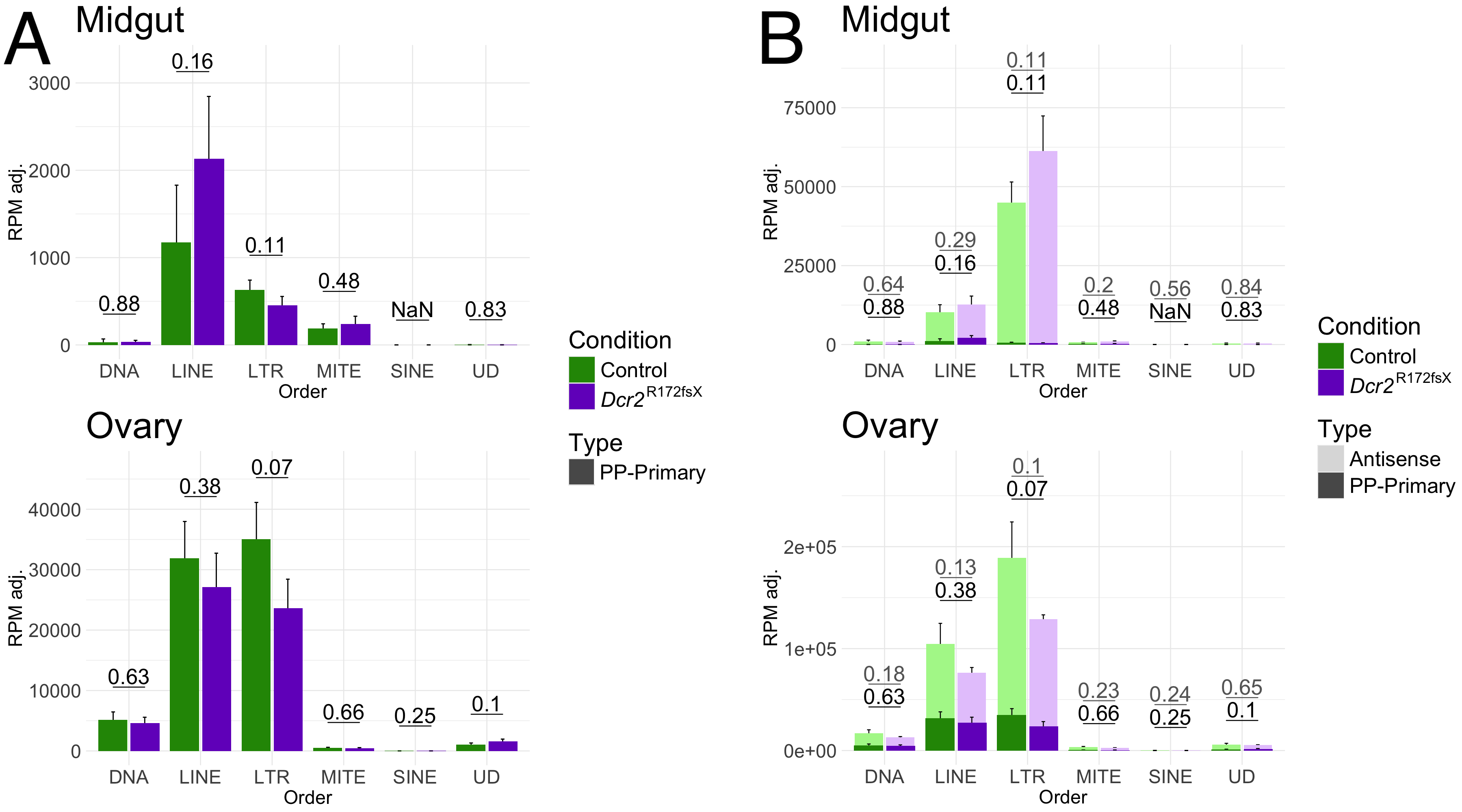

Supplement: Supplementary file 5 — Additional file 5: Figure S5 – Antisense and ‘ping-pong’-interacting primary piRNA abundance is equivalent between the two mosquito lines. (A) Amounts of putative primary piRNAs identified by 10nt overlap-based analysis of ping-pong signature (PP-primary) for each TE order for midgut (top) and ovary (bottom) samples. The error bars denote one standard deviation. The numbers above the bar plots indicate p-values obtained with Welch’s t-test. For SINE, no 10nt overlaps and thus no ‘ping-pong’-interacting primary piRNAs were detected in the midgut samples. (B) Amounts of antisense piRNA-sized reads for each TE order in midgut and ovary samples. The fraction of these that are considered putative primary piRNAs identified through 10nt overlap-based analysis of ping-pong signature (PP-primary) is highlighted in dark. The error bars denote one standard deviation. Grey numbers above the bars indicate the p-values obtained with Welch’s t-test for the amount of antisense piRNA-sized reads. Black numbers indicate p-values for putative ‘ping-pong’-interacting primary piRNAs [file 12915_2025_2225_MOESM5_ESM.png]

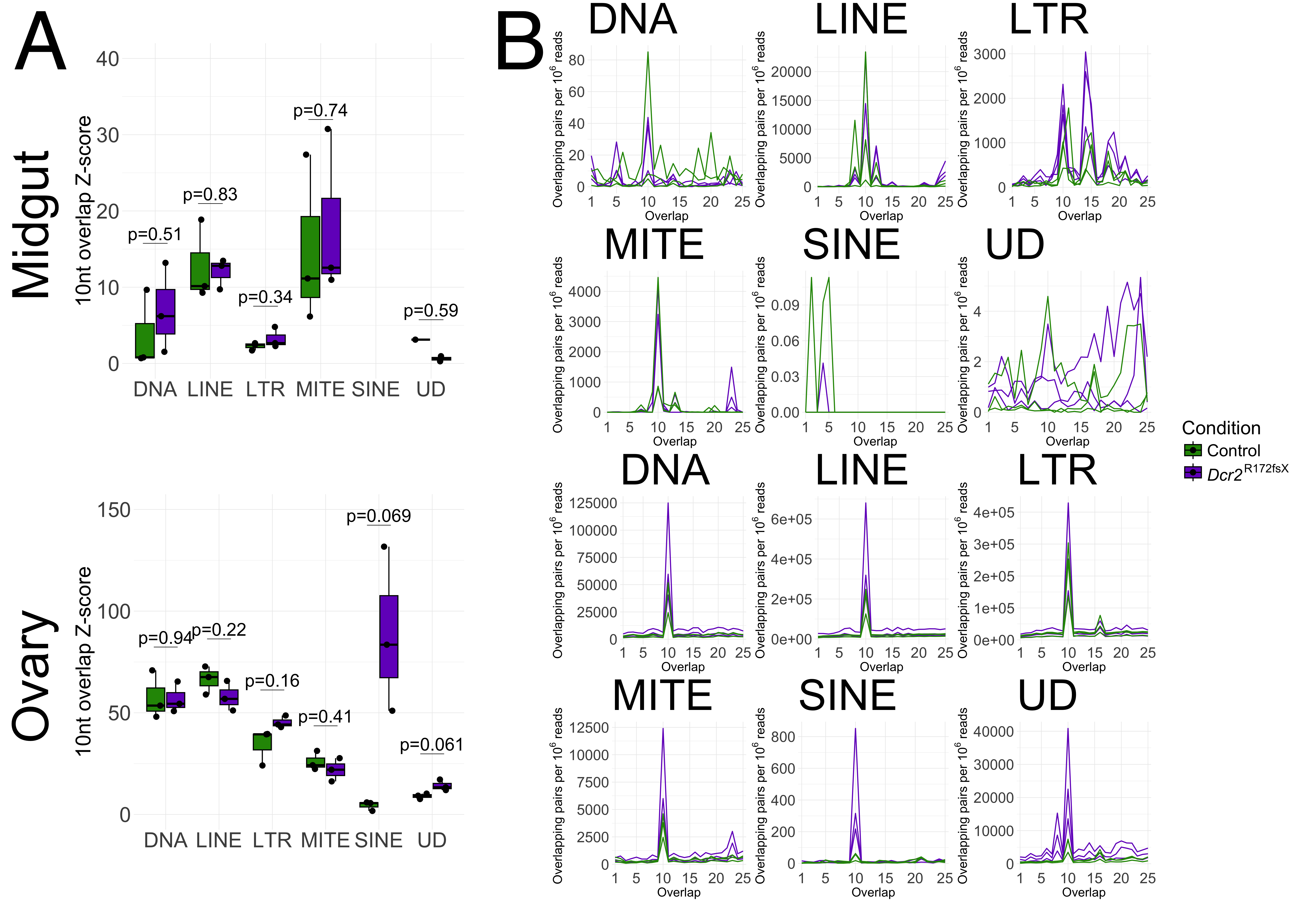

Supplement: Supplementary file 7 — Additional file 7: Figure S7 – Excluding the single locus contributing to the vast majority of 10nt overlaps for LTR transposons in the midgut abolishes the only difference between the Dcr2 mutant and control lines. (A) Box plots of 10nt overlap Z-scores among 26-30nt sense and antisense reads mapping to TEs in midguts (top) and ovaries (bottom). P-values indicated above the box pairs were obtained using Welch’s t-test. (B) Frequency of overlaps among sense and antisense reads by a given number of nt for midguts (top two rows) and ovaries (bottom two rows). Reads mapping to the copy of TE_0669_Gypsy that dominated the overlaps in control mosquito midguts were excluded from the plot for LTR transposons for the midgut samples. [file 12915_2025_2225_MOESM7_ESM.png]

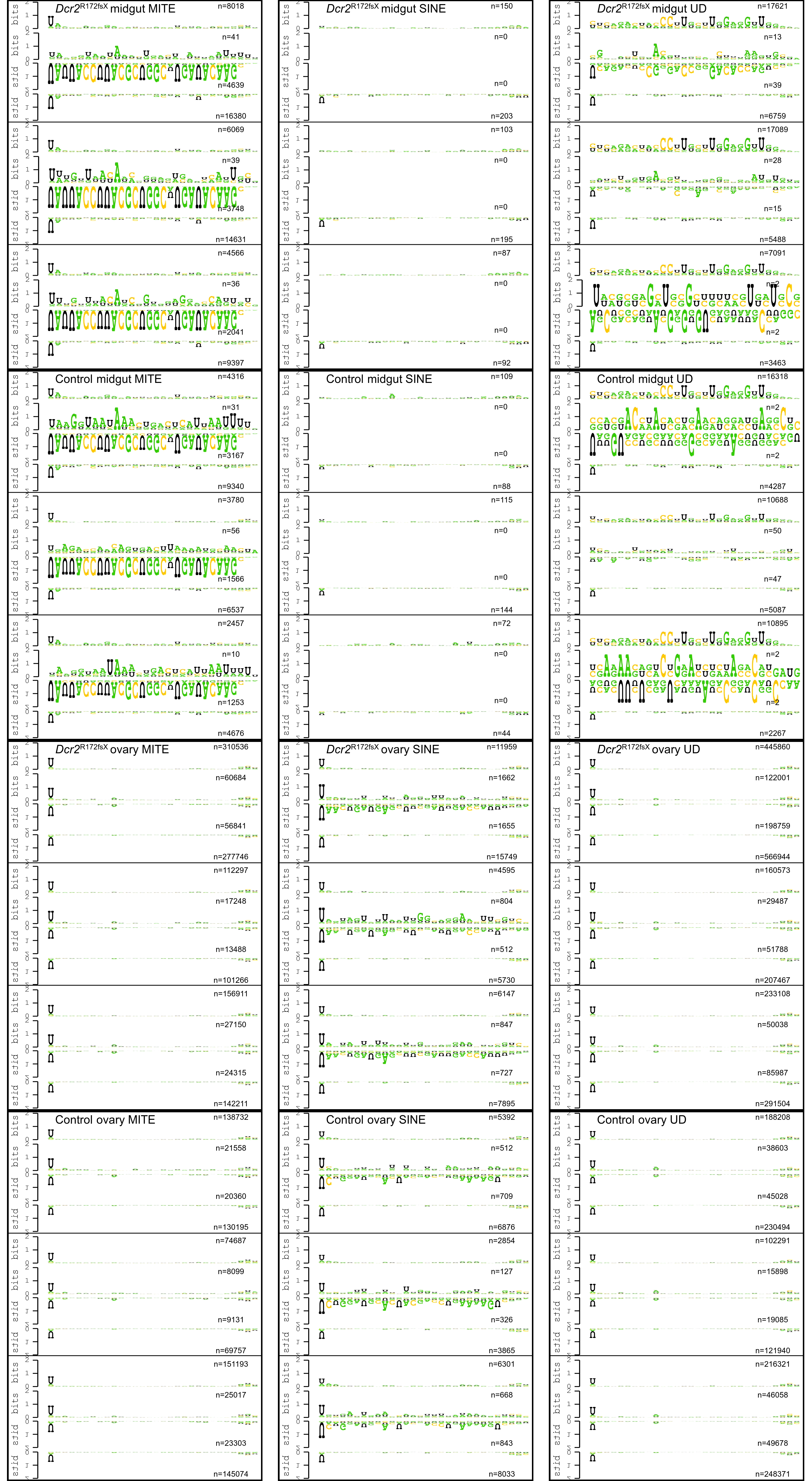

Supplement: Supplementary file 8 — Additional file 8: Figure S8 – No prominent 10 A bias for non-autonomous TE orders. Logo plots were constructed from 26-30nt reads mapping to the sense and antisense strands of TEs. Each column of logo plots in the figure corresponds to a TE order (MITE, SINE, UD = undetermined). The top half of the figure shows logo plots for the midgut samples, while the bottom half shows logo plots for the ovary samples. Within each half, the top half corresponds to samples from the Dcr2 mutant line, while the bottom corresponds to samples from control mosquitoes. Within each condition-organ-order partition, the three sets of four logo plots correspond to the three biological replicates. Each set is composed of four logo plots in the order top to bottom: 1) reads mapping to sense strand; 2) putative secondary piRNAs, i.e., reads mapping to sense strand and overlapping a putative primary piRNA in the secondary position (downstream of the corresponding putative primary piRNA) by 10 nt; 3) putative primary piRNAs engaged in the ‘ping-pong’ cycle, i.e., reads mapping to the antisense strand and overlapping a putative secondary piRNA in the primary position (upstream of the corresponding secondary piRNA) by 10 nt; 4) reads mapping to the antisense strand. The number of reads used to construct the logo is specified on the right side in each plot. [file 12915_2025_2225_MOESM8_ESM.png]

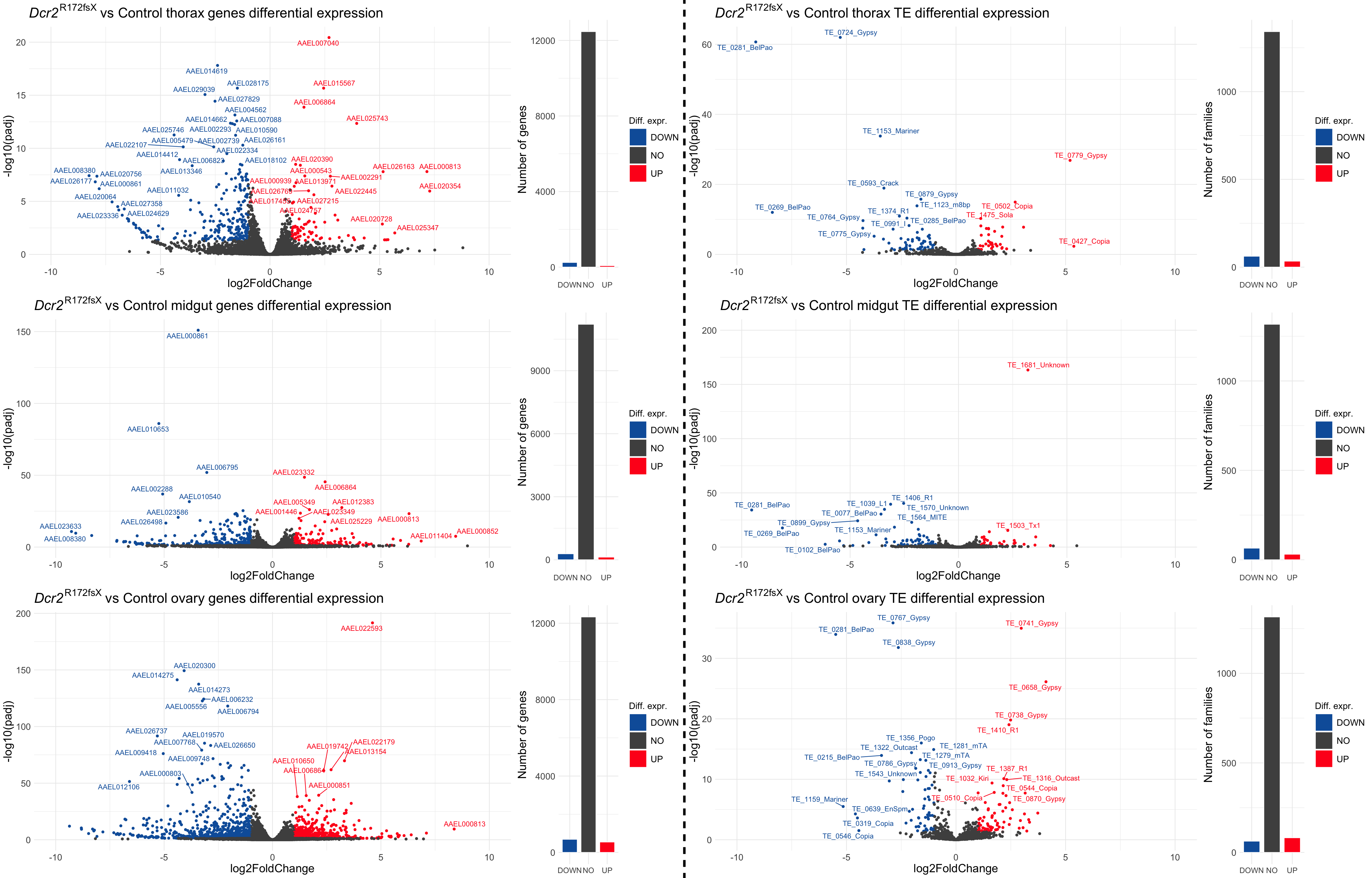

Supplement: Supplementary file 9 — Additional file 9: Figure S9 – Volcano plots and summary bar plots for differentially expressed genes and TE families show a wide perturbation of expression. Plots to the left of the partition correspond to genes, while plots to the right of the partition correspond to TEs. The three rows of plots correspond to the organs (ordered top to bottom): thorax, midgut, and ovary. The bar plots to the right of the volcano plot summarize the number of depleted (DOWN), non-differentially expressed (NO), and enriched (UP) genes or TEs in the Dcr2 mutant line relative to the control line. Genes or TE families with an absolute log2 fold-change > 1 and an adjusted p-value < 0.05 are colored according to the direction of their differential expression (red: enriched; blue: depleted; grey: not differentially expressed). [file 12915_2025_2225_MOESM9_ESM.png]

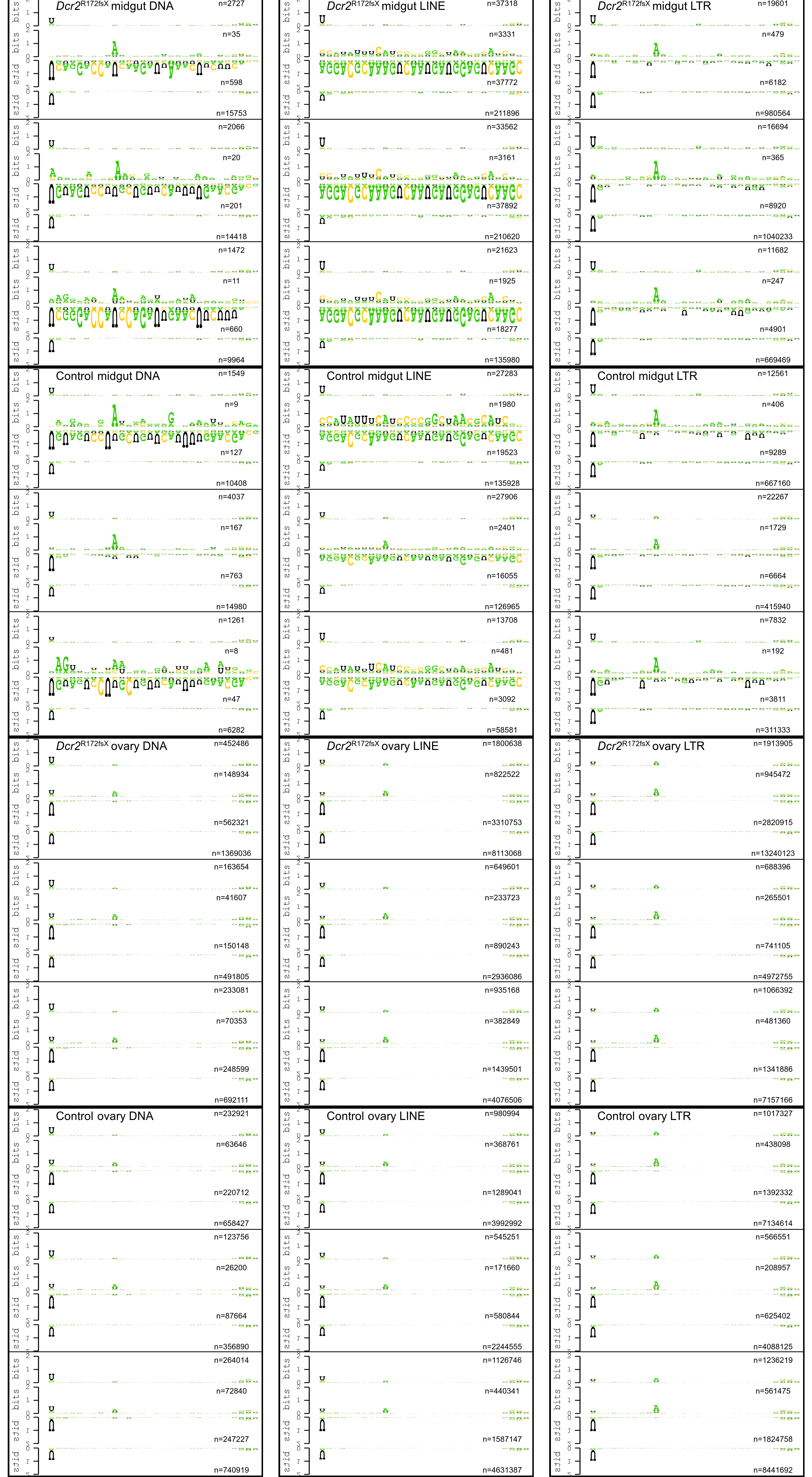

Supplement: Supplementary file 12 — Additional file 12: Figure S11 – Logo plots show 1U and 10 A bias in putative piRNAs only for certain autonomous TE orders. Logo plots were constructed from 26-30nt reads mapping to the sense and antisense strands of TEs. Each column of logo plots in the figure corresponds to a TE order (DNA, LINE, LTR). The plot is structured as in Additional file 8: Fig. S8 [file 12915_2025_2225_MOESM12_ESM.png]

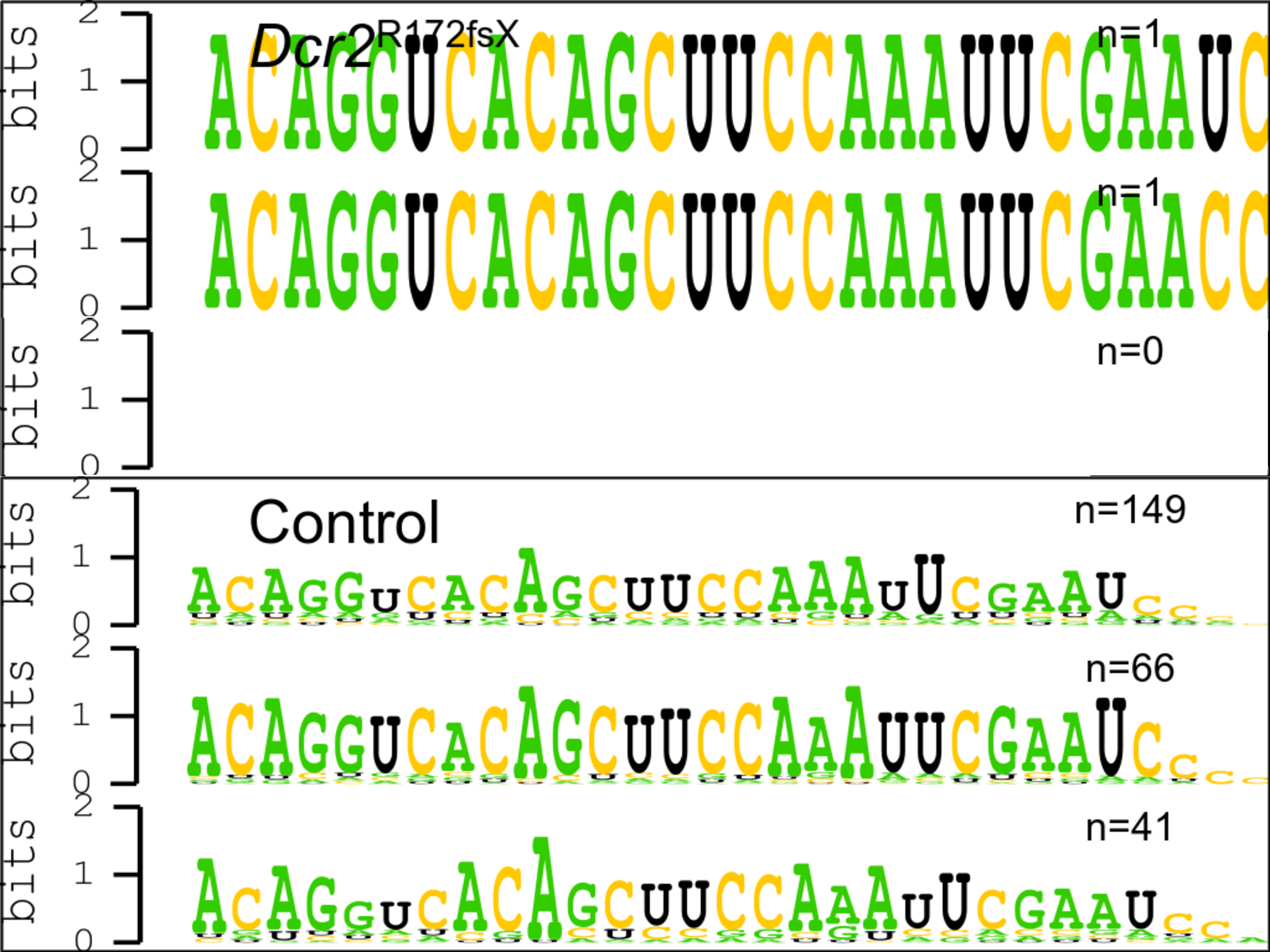

Supplement: Supplementary file 13 — Additional file 13: Figure S12 – Sequence composition of secondary piRNAs originating from the single locus mainly contributing to the 10nt overlaps among LTR transposons in the midgut of control mosquitoes. Logo plots derived from sequences mapping to the set of copies TE_0669_Gypsy_BTN, consisting of one single copy as seen in Additional file 6: Fig. S6B. Top half of figure shows logo plots from the three replicates of the Dcr2 mutant mosquitoes and bottom half shows the three replicates of the control mosquitoes. [file 12915_2025_2225_MOESM13_ESM.png]

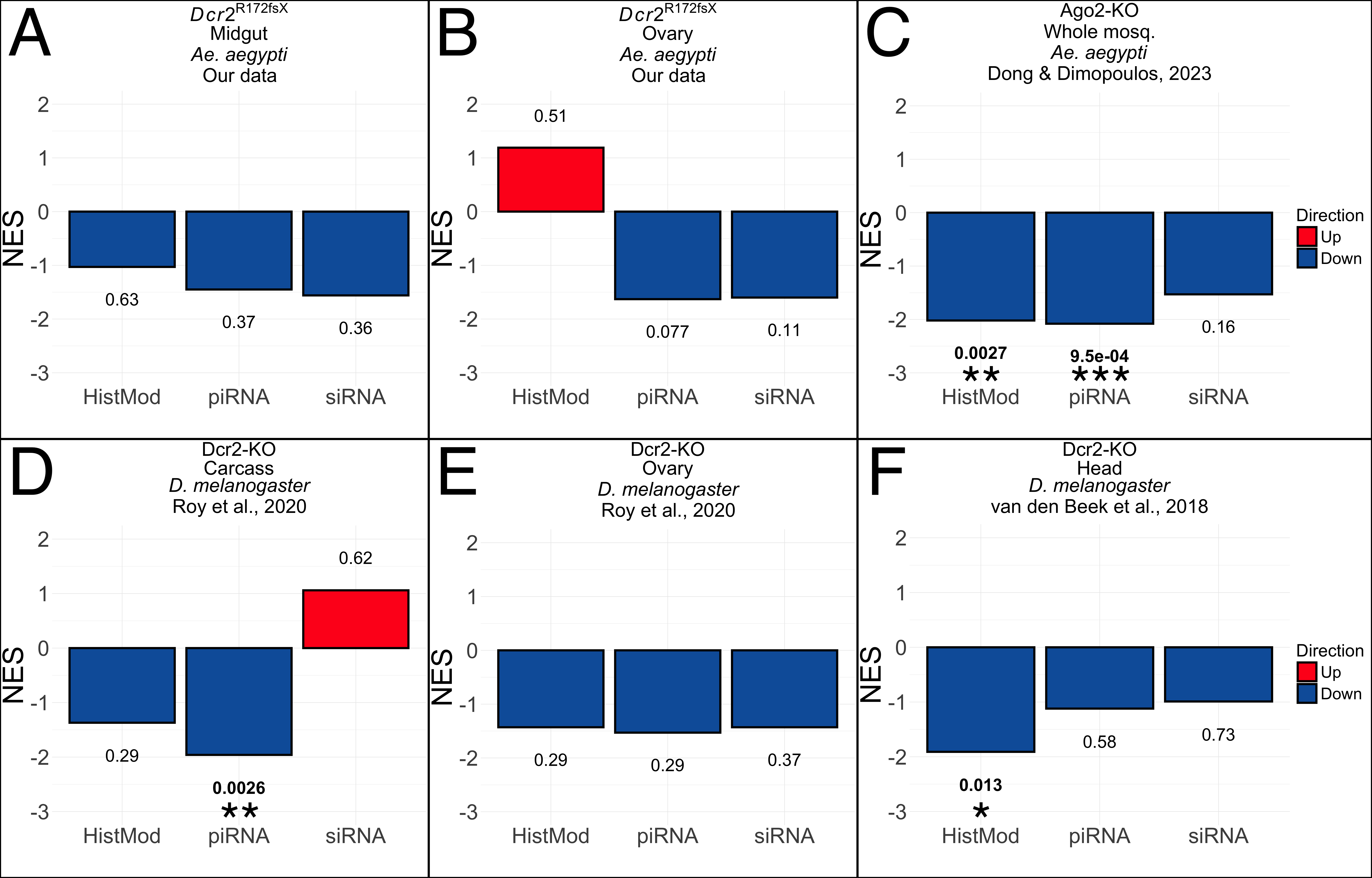

Supplement: Supplementary file 15 — Additional file 15: Figure S14 – Gene set enrichment analysis shows several cases of depleted pathways involved in TE regulation among all siRNA-pathway mutant datasets. The results of GSEAs for the the gene sets of interest with siRNA-, piRNA-, and histone modification-related (HistMod) functions for Ae. aegypti midguts (our data – A), ovaries (our data – B), and whole mosquitoes (Dong & Dimopoulos, [31] – C), and D. melanogaster carcasses (Roy et al. [37] – D), ovaries (Roy et al. [37] – E), and heads (Beek et al. [17] – F). The height of each bar represents the normalized enrichment score (NES), i.e., the relative, rank-based enrichment of the gene set compared to a random group of transcripts with the same size. Numbers above or below the bars indicate the false discovery rate for the enrichment (red bars) or depletion (blue bars) in the mutant relative to the wild-type control. Significant enrichments or depletions are highlighted in bold and with asterisks (*p < 0.05, **p < 0.01, ***p < 0.001). [file 12915_2025_2225_MOESM15_ESM.png]

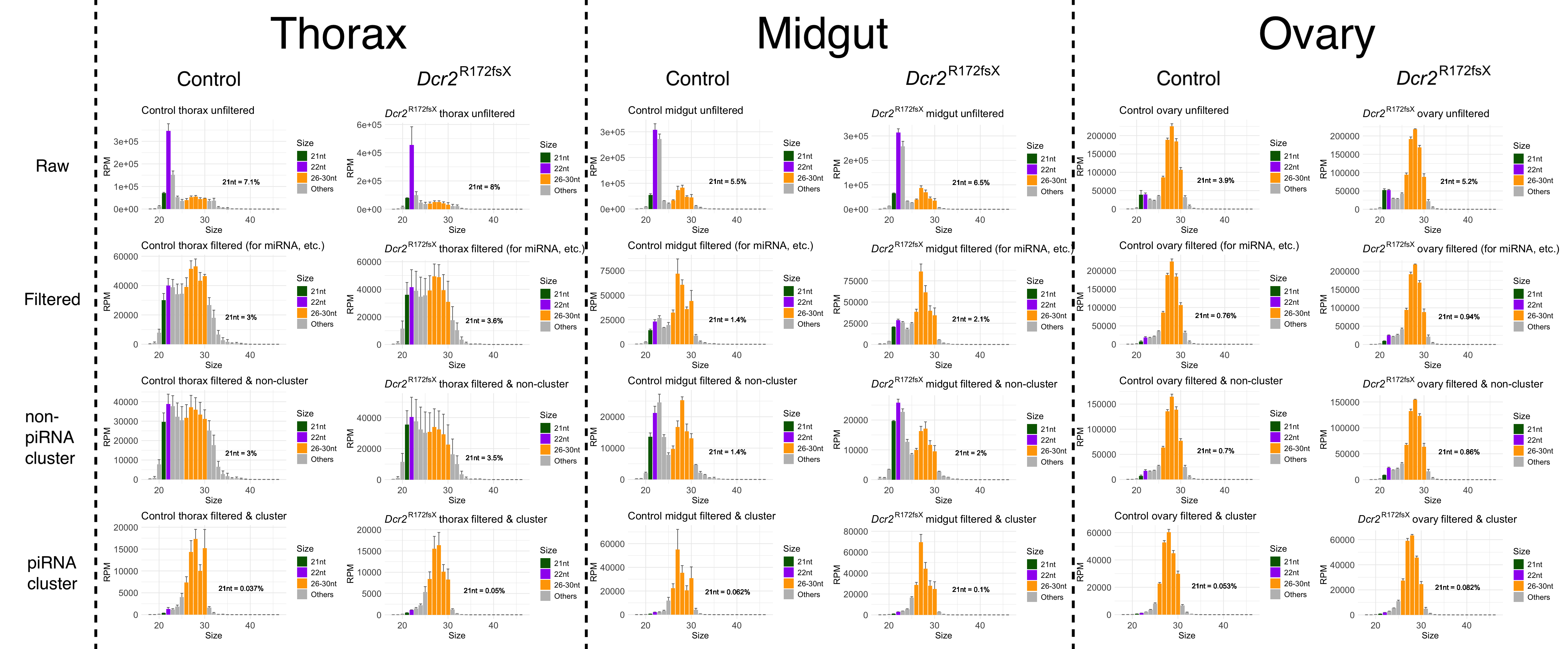

Supplement: Supplementary file 17 — Additional file 17: Figure S15 – Size distributions of small RNA reads following different levels of filtering. The plots are structured in three main columns – thorax samples are on the left, midgut samples in the center, and ovary samples on the right. Within each partitioned region, control mosquito libraries are shown on the left and Dcr2 mutant libraries are shown on the right. The rows correspond to reads subset based on filtering: First row – adapter-trimmed only (‘unfiltered/raw’), second row – filtered for reads mapping to miRNA, tRNA, snRNA, and snoRNA genes, third row – further filtered for reads mapping to annotated piRNA clusters, fourth row – only reads that map to piRNA clusters but not to any miRNA, tRNA, snRNA, or snoRNA genes. The percentage of 21nt reads is added to each plot. The error bars denote one standard deviation. When filtered for small RNA genes, a ‘block’ of reads can be seen in thorax samples, attributable to RNA degradation, while a piRNA-sized ‘hump’ can be seen most clearly in ovary samples, but also in midgut samples. Unfiltered reads from somatic tissues display a clear domination of the library by miRNA-sized reads, which are filtered away following intersection with annotated small RNA genes. [file 12915_2025_2225_MOESM17_ESM.png]

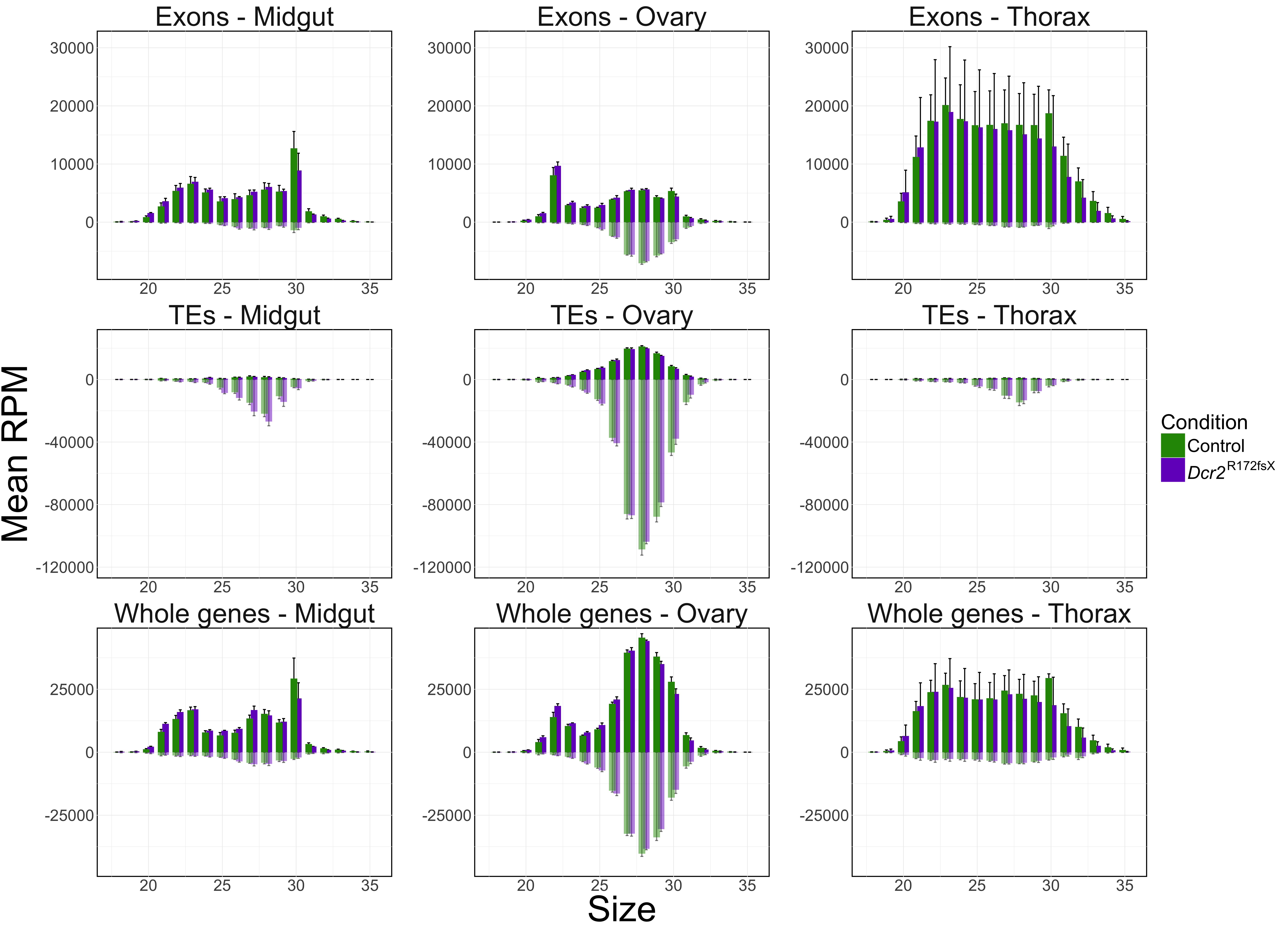

Supplement: Supplementary file 18 — Additional file 18: Figure S16 – Size distributions of small RNA reads mapping to exons, TEs, and whole genes. Size distributions of small RNA-seq reads filtered for miRNA, tRNA, snRNA, and snoRNA genes mapping to exons (top row), TEs (center row), and whole genes (exons and introns, bottom row) in the three different tissues – midgut (left column), ovary (center column), and thorax (right column). Reads mapping to the sense strand are shown with a positive RPM, reads mapping to the antisense strand are shown with a negative RPM. Thorax samples in both the Dcr2 mutant and the control mosquitoes show an overabundance of reads of various sizes mapping to the sense strand of genes and, in particular, the sense strand of exons. [file 12915_2025_2225_MOESM18_ESM.png]
